# Multi-target protective effects of *Agrimonia pilosa* Ledeb. against metabolic dysfunction-associated steatohepatitis in mice

**DOI:** 10.1080/13880209.2026.2632359

**Published:** 2026-03-09

**Authors:** Xinyi Fu, Jiawen You, Yunyi Yang, Shenglan Qi, Shiyu Yang, Xiaoxiao Qu, Yanting Shao, Ningwei Wang, Zhiying Wang, Yunhao Li, Min Zheng, Hongjie Yang, Jiajing Zhao, Xiaoli He, Yanming He

**Affiliations:** a Department of Endocrinology, Research Laboratory of Pharmacy, Center of Experimental Animals, Clinical Research Institute of Integrative Medicine, Yueyang Hospital of Integrated Traditional Chinese and Western Medicine, Shanghai University of Traditional Chinese Medicine, Shanghai, China; b Key Laboratory of Liver and Kidney Diseases (Ministry of Education), Institute of Liver Diseases, Shuguang Hospital Affiliated to Shanghai University of Traditional Chinese Medicine, Shanghai, China; c Shanghai Putuo District Traditional Chinese Medicine Hospital, Shanghai, China

**Keywords:** MASH, *Agrimonia pilosa*, agrimol B, hepatic steatosis, inflammatory response

## Abstract

**Context:**

*Agrimonia pilosa* Ledeb. (AP), a traditional herbal medicine rich in flavonoids, phenolics, triterpenoids, and glycosides, has been widely used for hepatic injury and metabolic disorders.

**Objective:**

This study integrated *in vivo* and *in vitro* experiments, UHPLC-HRMS profiling, network pharmacology, and molecular simulation to elucidate the bioactive constituents and mechanisms of AP against metabolic dysfunction-associated steatohepatitis (MASH).

**Materials & Methods:**

Therapeutic efficacy was evaluated using a MASH mouse model, AML12 hepatocytes, and RAW264.7 macrophages. Active constituents were identified by UHPLC-HRMS, and potential targets were predicted via SwissTargetPrediction and GEO databases, followed by PPI network construction, GO/KEGG enrichment analysis, molecular docking, and molecular dynamics simulation.

**Results:**

AP markedly reduced body weight, liver index, and serum AST, ALT, TG, TC, and LDL-c levels, and attenuated hepatic steatosis, inflammation, and fibrosis. In AML12 cells, AP suppressed lipogenesis by downregulating SREBP-1c, FASN, and SCD1, while promoting fatty acid β-oxidation through CPT1A upregulation. In RAW264.7 macrophages, AP inhibited LPS-induced expression of TNF-α, IL-1β, and IL-6. A total of 83 active constituents and 25 key targets were identified, with HMGCR and AXL emerging as hub nodes. Agrimol B (AGB) exhibited favorable binding affinity and structural stability toward both targets. Mechanistically, AGB inhibited HMGCR, reduced SREBP-2 nuclear translocation, and enhanced LXRα/β-mediated cholesterol efflux, maintaining hepatic cholesterol homeostasis.

**Discussion and Conclusion:**

These findings demonstrate that AP ameliorates MASH through coordinated regulation of lipid metabolism, inflammatory suppression, and collagen deposition, with AGB representing a promising bioactive candidate warranting further investigation.

## Introduction

Metabolic dysfunction-associated steatotic liver disease (MASLD) is one of the leading causes of chronic liver disease worldwide (Kanwal et al. 2024). Its progressive subtype, metabolic dysfunction-associated steatohepatitis (MASH), is characterized by hepatic inflammation and disease progression. The prevalence of MASLD and MASH has increased markedly over the past decade, with the global prevalence of MASH in the general population estimated at approximately 5.5% between 2016 and 2019 (Younossi 2023). Histologically, MASH is predominantly localized to zone 3 of the hepatic acinus, characterized by macrovesicular and medium-sized steatosis, lobular necroinflammation, and mild portal inflammation (Li et al. 2023). Epidemiological data indicate that the global prevalence of MASLD/MASH in adults increases with body mass index (BMI) (Dong et al. 2024). Lifestyle-related factors, particularly caloric excess, dietary patterns, and alcohol consumption, play pivotal and interrelated roles in disease onset and progression. Excessive energy intake and poor dietary quality promote hepatic lipid accumulation, while alcohol exposure may synergize with metabolic dysfunction to exacerbate liver injury and influence therapeutic responses (Valenzuela et al. 2026). Consequently, international multidisciplinary expert consensus emphasizes that dietary intervention constitutes a cornerstone strategy for the prevention and management of MASLD (Zeng et al. 2024). Although some patients with simple hepatic steatosis may remain stable over extended periods, a substantial proportion progress to MASH, thereby significantly increasing the risk of fibrosis, cirrhosis, hepatocellular carcinoma, liver transplantation, and liver-related mortality (Golabi et al. 2024). MASH is strongly associated with insulin resistance, obesity, dyslipidemia, hypertension, and diabetes (Hagström et al. 2024). The pathogenesis of MASH remains incompletely understood. The ‘multiple-hit’ hypothesis posits that multiple parallel insults act on genetically susceptible individuals to induce MASH. These insults include insulin resistance, adipokines, nutritional factors, gut microbiota, as well as genetic and epigenetic factors (Buzzetti et al. 2016). Due to the complex pathogenesis of MASH, current pharmacological treatments have failed to adequately meet clinical needs (Zhi et al. 2024).

*Agrimonia pilosa* Ledeb. (*A. pilosa*, AP), a perennial herb of the Rosaceae family, is the dried aerial part of the plant, widely distributed across the northern temperate zone, tropical highlands, and Latin America (Wen et al. 2022). Traditionally, AP has been used for its hemostatic, astringent, anti-malarial, anti-dysenteric, tonic, and spleen-strengthening properties. Pharmacological studies have shown that AP possesses therapeutic potential in the treatment of inflammation, hepatic injury, diabetes, obesity, myocardial injury, and cancer (Şener et al. 2022; Huang et al. 2023; Liu et al. 2024). However, whether AP can ameliorate MASH and the underlying mechanisms remain unclear. AP contains diverse bioactive compounds, including flavonoids, phenolics, triterpenoids, and glycosides, many of which exhibit therapeutic effects against metabolic disorders. Recent studies have demonstrated that flavonoids extracted from AP regulate insulin signaling pathways and adipose tissue metabolism to improve diabetes (Guo et al. 2023). Furthermore, several flavonoids in AP, such as quercetin, apigenin, and kaempferol, exert significant anti-inflammatory effects (Park et al. 2024). In the present study, we aimed to evaluate the pharmacological efficacy of AP in the treatment of MASH and to identify its major active constituents.

A MASH animal model was established in C57BL/6 mice. Pharmacological effects of *A. pilosa* extract were assessed through body weight, liver weight, liver function, serum lipids, hepatic fat accumulation, and histopathological changes. *In vitro*, normal murine hepatocyte AML12 cells and RAW264.7 macrophages were employed to construct MASH lipid overload and inflammatory models, respectively. The efficacy of AP was evaluated by examining hepatic pathology, expression of lipid metabolism-related genes, macrophage activation, and pro-inflammatory cytokine gene expression. Given the complexity of multi-component herbal medicines, elucidating their pharmacological mechanisms remains challenging. With the increasing use of bioinformatics tools in disease research and drug discovery, network pharmacology has been widely applied to investigate the therapeutic potential of natural products, enabling a systems-level evaluation of multi-component, multi-target, and multi-pathway interactions. Molecular docking techniques are employed to predict conformational binding sites and assist in drug screening, while molecular dynamics simulations provide insights into the dynamic interactions between bioactive compounds and target proteins (Lu et al. 2024; Yuan et al. 2024).

In this study, we combined *in vivo* and *in vitro* experimental validation with liquid chromatography-mass spectrometry (HPLC-MS) to confirm the therapeutic efficacy of AP against MASH. Moreover, we employed bioinformatics analysis, network pharmacology, molecular docking, and molecular dynamics simulation to explore the active constituents of AP and their potential molecular targets in the treatment of MASH.

## Materials and methods

### Drug sources

*Agrimonia pilosa* Ledeb. (*A. pilosa*, AP) (Cat. No. 220123) was purchased from Hongqiao Chinese Herbal Decoction Pieces Co., Ltd. (Shanghai, China). Pioglitazone (Cat. No. 112529-15-4, purity ≥ 98%) was obtained from Efebio Biotechnology Co., Ltd. (Chengdu, China), and celecoxib (Cat. No. B58349-10mg, purity ≥ 98%) was purchased from Yuanye Biotechnology Co., Ltd. (Shanghai, China).

A total of 500 g of AP decoction pieces were soaked in 10 volumes of distilled water at room temperature for 30 min, heated to boiling, and decocted for 30 min. The decoction was filtered, and the filtrate was collected. The residue was decocted again in 8 volumes of distilled water for 30 min and filtered. Both filtrates were combined, filtered through gauze while hot, and concentrated under reduced pressure to a thick paste, which was stored at −20 °C until further use.

### Identification of AP constituents by UHPLC-Q-Exactive Orbitrap HRMS

Compounds in AP were analyzed using UHPLC-Q-Exactive Orbitrap HRMS (Thermo Fisher Scientific Inc., Grand Island, NY, USA). Chromatography was performed on a Thermo Scientific Dionex Ultimate 3000 HPLC system, controlled by Chromeleon 7.2 software. The cooled autosampler was maintained at 10 °C and protected from light, and the column heater was set at 45 °C. Separation was carried out on a Waters ACQUITY UPLC BEH C18 column (2.1 × 100 mm, 1.7 μm). The mobile phase consisted of A (methanol) and B (0.1% formic acid), at a flow rate of 0.3 mL·min^−1^, with gradient elution as follows: 0–2.0 min (4% A), 2.0–6.0 min (4–12% A), 6.0–38.0 min (12–70% A), 38.0–38.5 min (70% A), 38.5–39.0 min (70–95% A), 39.0–43.0 min (95% A), 43.0–45.0 min (4% A). The injection volume was 2 μL.

Mass spectrometry was performed using a Q-Orbitrap system with a heated electrospray ionization source connected to the UHPLC system, controlled by Xcalibur 4.1 software. Analyses were performed in negative ionization mode. The optimized MS parameters were: capillary temperature, 320 °C; sheath gas (N_2_), 35 arbitrary units; auxiliary gas (N_2_), 10 arbitrary units. The scanning mode was full MS, with a scan range of 80–1200 m/z, targeting 100 compounds for both qualitative and quantitative analysis. The maximum injection time (IT) was 200 ms, resolution 70,000 FWHM (m/z/s), and automatic gain control (AGC) target 1.0e^6^.

### Experimental animals, diets, and treatment protocol

Twenty-four 5-week-old male C57BL/6 mice (15–20 g) were obtained from Jihui Laboratory Animal Breeding Co., Ltd. (Shanghai, China). Mice were acclimated for one week at the animal facility of Yueyang Hospital of Integrated Traditional Chinese and Western Medicine, Shanghai University of Traditional Chinese Medicine (license No. SYXK2018-0040). After acclimation, mice were randomly assigned to the following experimental groups: the normal control group (**Normal**, *n* = 6) and the high-fat, high-carbohydrate, cholesterol diet–fed group (HFHCD, *n* = 18). The control group received a standard chow diet and water (the composition of the normal diet is shown in Supplementary Table 4), whereas the HFHCD group was continuously fed the HFHCD diet (diet composition is provided in Supplementary Tables 5–6, and the energy density comparison between the diets is summarized in Supplementary Table 7) and high-fructose water (23.1 g/L fructose and 18.9 g/L glucose) for 24 weeks to induce MASH.

After 20 weeks of HFHCD feeding, mice in the HFHCD group were further randomly subdivided into three groups (*n* = 6 per group): the HFHCD group (**HFHCD**, continued HFHCD feeding without intervention), the HFHCD + AP group (**AP**), and the HFHCD + PGZ group (**PGZ**). Mice in the AP group received AP extract by oral gavage, whereas mice in the HFHCD + PGZ group were treated with pioglitazone hydrochloride as a positive control. All interventions were administered while maintaining HFHCD feeding until the end of the 24-week experimental period.

### Reagents and drugs

Fetal bovine serum (FBS; Cat. No. A3160901), Dulbecco’s Modified Eagle Medium (DMEM; Cat. No. 11995065), ITS supplement (ITS-G; Cat. No. 41400045), penicillin-streptomycin solution (Cat. No. 15140122), and 0.25% trypsin (Cat. No. 25200056) were purchased from Gibco (Thermo Fisher Scientific Inc., MA, USA). Phosphate-buffered saline (PBS; Cat. No. C0221A), lipopolysaccharide (LPS; Cat. No. S1732-5mg), dexamethasone (Cat. No. ST1258-50mg), dimethyl sulfoxide (DMSO; Cat. No. ST2335-25ml), Hematoxylin and Eosin staining kit (Cat. No. C0105M), Oil Red O staining kit (Cat. No. C0157S), DAPI solution (Cat. No. C1002), and Cell Counting Kit-8 (CCK-8; Cat. No. C0038) were obtained from Beyotime (Shanghai, China). Reverse transcription (Cat. No. BL699A) and quantitative PCR kits(Cat. No. BL698A) were from Lanjie Ke (Beijing, China). TRIzol reagent (Cat. No. N065), aspartate aminotransferase (AST; Cat. No. C010-1-1) kit, alanine aminotransferase (ALT; Cat. No. C009-1-1) kit, triglyceride (TG; Cat. No. A110-1-1) kit, and total cholesterol (TC; Cat. No. A111-1-1) kit were purchased from Jiancheng Institute of Biotechnology (Nanjing, China). Ethanol (Cat. No. XW00641752), xylene (Cat. No. 10023418), oleic acid (Cat. No. 30138518), palmitic acid (Cat. No. 30139518) were purchased from China National Pharmaceutical Group Co., Ltd. (Beijing, China).

### Hematoxylin and eosin (HE) staining of liver tissue

Paraffin-embedded liver tissues were prepared after fixation in 4% paraformaldehyde for 3 days. Samples were dehydrated through graded ethanol, cleared with xylene, and embedded in paraffin at 60 °C. Sections (4 μm) were cut, floated in a 40 °C water bath, and mounted on slides. Following deparaffinization and rehydration, slides were stained sequentially with hematoxylin and eosin, dehydrated, cleared, and mounted.

### Oil Red O staining of liver tissue

Frozen liver sections (8 μm) were mounted on slides, fixed, and stained with Oil Red O working solution for 20 min, followed by hematoxylin counterstaining for 5 min. Slides were mounted with glycerol gelatin for microscopic examination.

### Sirius Red staining of liver tissue

Paraffin sections were deparaffinized, rehydrated, and stained with Sirius Red for 1 h, followed by differentiation with 0.5% acetic acid for 20 s. Sections were dehydrated in ethanol, cleared in xylene, and mounted with neutral resin.

### Measurement of serum and hepatic lipids and enzymes

Serum TG, TC, LDL, ALT, and AST were measured using commercial kits (Nanjing Jiancheng Bioengineering Institute, Nanjing, China). Blood samples were allowed to stand for 30 min and centrifuged at 3000 rpm for 10 min to obtain serum, which was immediately analyzed. Hepatic TG and TC contents were similarly determined using the corresponding kits.

### Cell culture

Murine macrophages (RAW264.7; CVCL_0493; Cat No. SCSP-5036) and hepatocytes (AML12; CVCL_0140; Cat No. SCSP-550) were obtained from the Chinese Academy of Sciences Cell Bank (Shanghai, China). RAW264.7 cells were cultured in DMEM containing 10% FBS and 1% penicillin-streptomycin. AML12 cells were maintained in DMEM supplemented with 10% FBS, 1% penicillin-streptomycin, 10 μg/mL insulin, 5.5 μg/mL transferrin, 5 ng/mL selenium, and 40 ng/mL dexamethasone. All cells were cultured at 37 °C in 5% CO_2_.

### Establishment of *in vitro* MASH models

RAW264.7 cells were treated with 1 μg/mL LPS for 24 h to establish an *in vitro* MASH inflammatory model (Merighi et al. 2021). AML12 cells were treated with 300 μM free fatty acids (FFAs, oleic acid:palmitic acid = 2:1) for 24 h to induce lipid overload (Wu et al. 2024).

### CCK-8 assay for cell viability

AML12 and RAW264.7 cells were treated with varying concentrations of *A. pilosa* extract (6.4 μg/mL to 100 mg/mL) for 24 h. Cell viability was subsequently evaluated using the Cell Counting Kit-8. Cells (5 × 10^3^/well) were seeded in 96-well plates and cultured for 24 h. After PBS wash, cells were treated with compound-containing or control medium; blank wells contained medium only. Following 24 h incubation, 10 μL CCK-8 was added, incubated for 2 h, and absorbance at 450 nm was measured to determine viability.

### Oil Red O staining of hepatocytes

AML12 cells grown in 24-well plates were fixed with 4% paraformaldehyde for 15 min at room temperature, and stained with Oil Red O working solution for 10 min. After washing with 60% isopropanol and distilled water, nuclei were counterstained with hematoxylin for 1 min. Lipid droplets were visualized and imaged using an inverted microscope (Olympus, Japan).

### Immunofluorescence assay

RAW264.7 cells were cultured in 48-well plates and treated for 24 h. Cells were fixed with 4% paraformaldehyde, permeabilized with 0.2% Triton X-100, blocked with 5% BSA, and incubated with anti-CD11b antibody overnight (information of antibodies in Supplementary Table 1). After washing, cells were incubated with Cy3-conjugated secondary antibody, counterstained with DAPI, and mounted. Images were acquired using a confocal laser scanning microscope (Olympus FV10C-W3, Japan).

### Real-time PCR (RT-PCR)

RAW264.7 and AML12 cells were cultured and treated in 6-well plates. Total RNA was extracted with TRIzol and reverse-transcribed into cDNA. Quantitative PCR was performed using specific primers (Supplementary Table 2; designed and synthesized by Takara Bio, Dalian, China).

### Bioinformatics analysis and target screening of AP against MASH

Active compounds of AP identified in Section 2.1 were queried from PubChem to obtain SMILES formats. Drug-likeness was predicted using SwissADME, applying Lipinski ≤ 1, Bioavailability Score ≥ 0.17, and high GI absorption as screening criteria. Targets were predicted with SwissTargetPrediction (probability > 0). MASH-related differentially expressed genes (DEGs) were obtained from GEO dataset GSE126848 and analyzed with the R package limma. Venn diagrams were constructed using Jvenn to identify overlapping targets between AP and MASH DEGs. Protein-protein interaction (PPI) networks were established using STRING and visualized in Cytoscape. GO and KEGG enrichment analyses were performed in R.

### Molecular docking

Active compounds and key protein targets were downloaded from PubChem and PDB, respectively. Docking was performed using AutoDock after receptor preparation (removal of water molecules and ligands, hydrogenation, and charge addition). Visualization of docking results was carried out with PyMOL.

### Molecular dynamics simulation

Protein-ligand complexes were processed using PyMOL and simulated with Gromacs 2022.6 with the CHARMM36 force field. Systems were solvated in a water box and neutralized with counterions. Energy minimization, NVT and NPT equilibrations were performed, followed by 100 ns production runs at 300 K and 1 bar. RMSD, Rg, RMSF, SASA, hydrogen bond numbers, and free energy landscapes were analyzed.

### Statistical analysis

Data were expressed as mean ± standard deviation (SD). Statistical analyses were performed with SPSS 22.0. Normality and homogeneity of variance were tested prior to one-way ANOVA. Post hoc analyses were conducted using Tukey’s test for homogeneous variances or Games-Howell for heterogeneous variances. A P-value < 0.05 was considered statistically significant.

## Results

### AP significantly ameliorates HFHCD-induced steatohepatitis-like liver injury in mice

**Figure 1.**
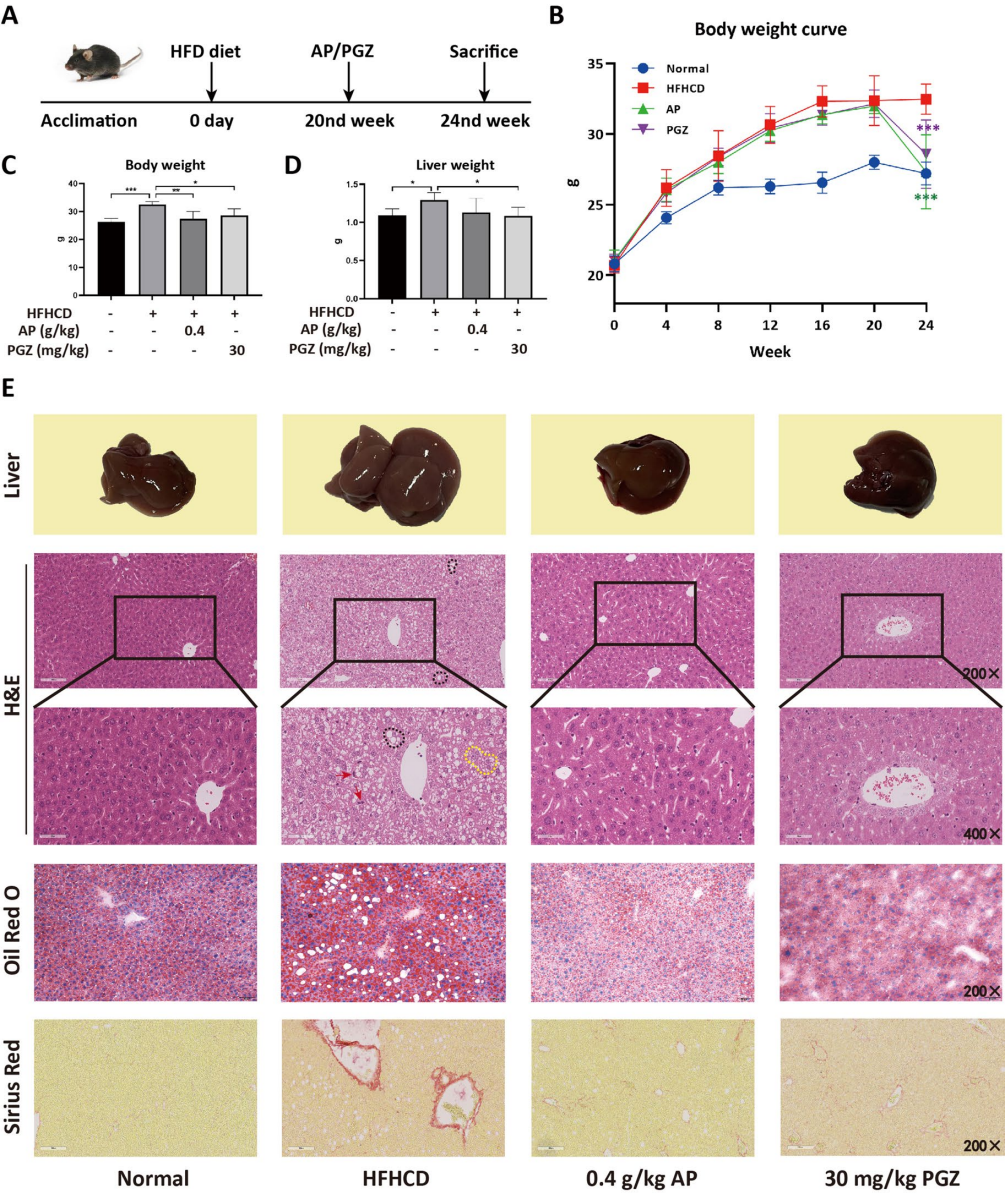
The therapeutic effects of *A. pilosa* on an HFHCD-induced MASH mouse model. A MASH animal model was established by feeding male C57BL/6 mice a high-fat, high-cholesterol diet (HFHCD) for 24 weeks, followed by a 4-week treatment with either 0.4 g/kg *A. pilosa* or 30 mg/kg pioglitazone (PGZ) (A). Panels (B) and (C) depict the body weight changes in the mice during and after treatment, while panel (D) shows post-treatment liver weights. Histological features of liver tissue following *A. pilosa* treatment were assessed by liver gross morphology, H&E staining, Oil Red O staining, and Sirius Red staining (magnification, 200× and 400×) (E). Compared with the model group, significant differences are indicated as follows: *p < 0.05, **p < 0.01, ***p < 0.001. Macrovesicular steatosis (black dashed outlines), microvesicular steatosis (yellow dashed outlines), and inflammatory foci (red arrows) are indicated in H&E-stained sections. Oil Red O staining shows hepatic lipid accumulation, and Sirius Red staining highlights collagen deposition.

In the HFHCD-fed mouse model exhibiting hepatic steatosis and liver inflammation, animals were randomly divided into the HFHCD group, AP group, and PGZ group at week 20, receiving daily oral administration of 0.9% saline, 0.4 g/kg AP, or 30 mg/kg PGZ for 4 weeks (Figure 1A). During the initial 20 weeks of HFHCD feeding, except for the normal control group, body weight of all remaining mice increased significantly, with no significant differences among groups. As treatment progressed, AP markedly reduced body weight in model mice (*p* < 0.001) (Figure 1B,C). Morphologically, livers from the HFHCD group appeared enlarged, swollen, and pale, whereas AP and PGZ treatment restored liver appearance closer to the normal controls (Figure 1E). Regarding liver weight, PGZ significantly reduced liver weight, while no obvious reduction was observed in the AP group (*p* < 0.05) (Figure 1D). Histopathological analysis revealed pronounced inflammatory infiltration and ballooning degeneration in model mice, which were substantially alleviated following AP treatment (Figure 1E). Oil Red O staining demonstrated a marked reduction of hepatic lipid droplets in both AP and PGZ groups (Figure 1E). Sirius Red staining further showed attenuated hepatic fibrosis in the treatment groups (Figure 1E).

**Figure 2.**
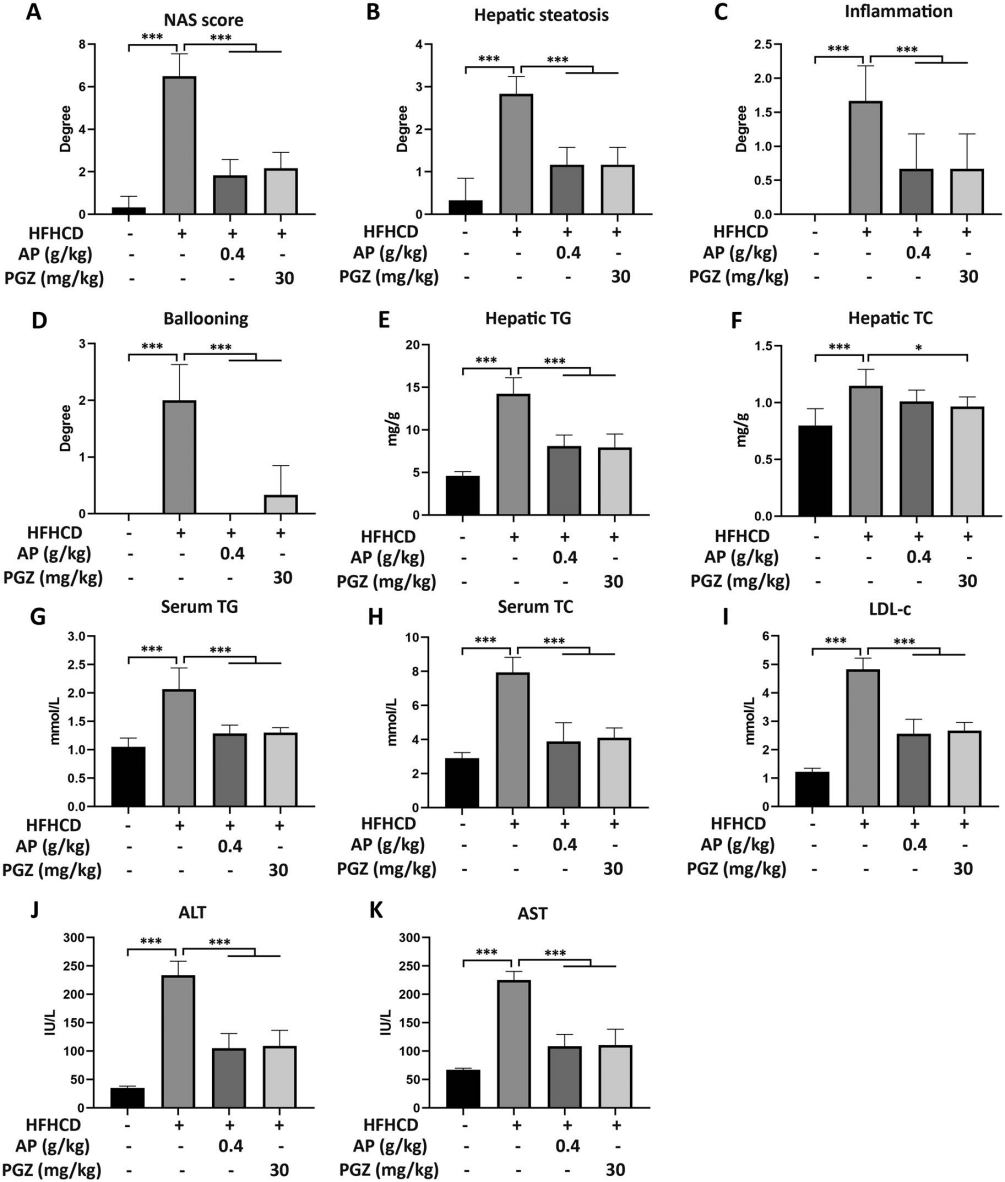
Efficacy evaluation of *A. pilos*a in the MASH animal model. NAS scores (A) were calculated, including assessments of hepatosteatosis (B), lobular inflammation (C), and hepatocyte ballooning (D). Scoring criteria were as follows: (1) Hepatosteatosis (<5%, score 0; 5-33%, score 1; 33-66%, score 2; >66%, score 3); (2) lobular inflammation (none, score 0; <2 foci per 200× field, score 1; 2–4 foci per 200× field, score 2; >4 foci per 200× field, score 3); (3) hepatocyte ballooning (none, score 0; rare, score 1; extensive, score 2). Hepatic levels of TG (E) and TC (F) were determined. Serum levels of TG (G), TC (H), and LDL-c (I) were measured, along with ALT (J) and AST (K). Comparisons with the model group are indicated as follows: ***p < 0.001.

NAFLD activity score (NAS) assessment indicated that AP and PGZ significantly reduced steatosis, lobular inflammation, and hepatocyte ballooning compared with the HFHCD group (*p* < 0.001) (Figure 2A–D). Furthermore, both treatments significantly decreased serum levels of TG, TC, LDL-c, ALT, and AST (*p* < 0.001) (Figure 2G–K). Intrahepatic lipid quantification revealed that AP and PGZ markedly reduced hepatic TG and TC levels (*p* < 0.001) (Figure 2E,F).

### AP attenuates steatosis and restores lipid homeostasis in FFA-induced AML12 cells

**Figure 3.**
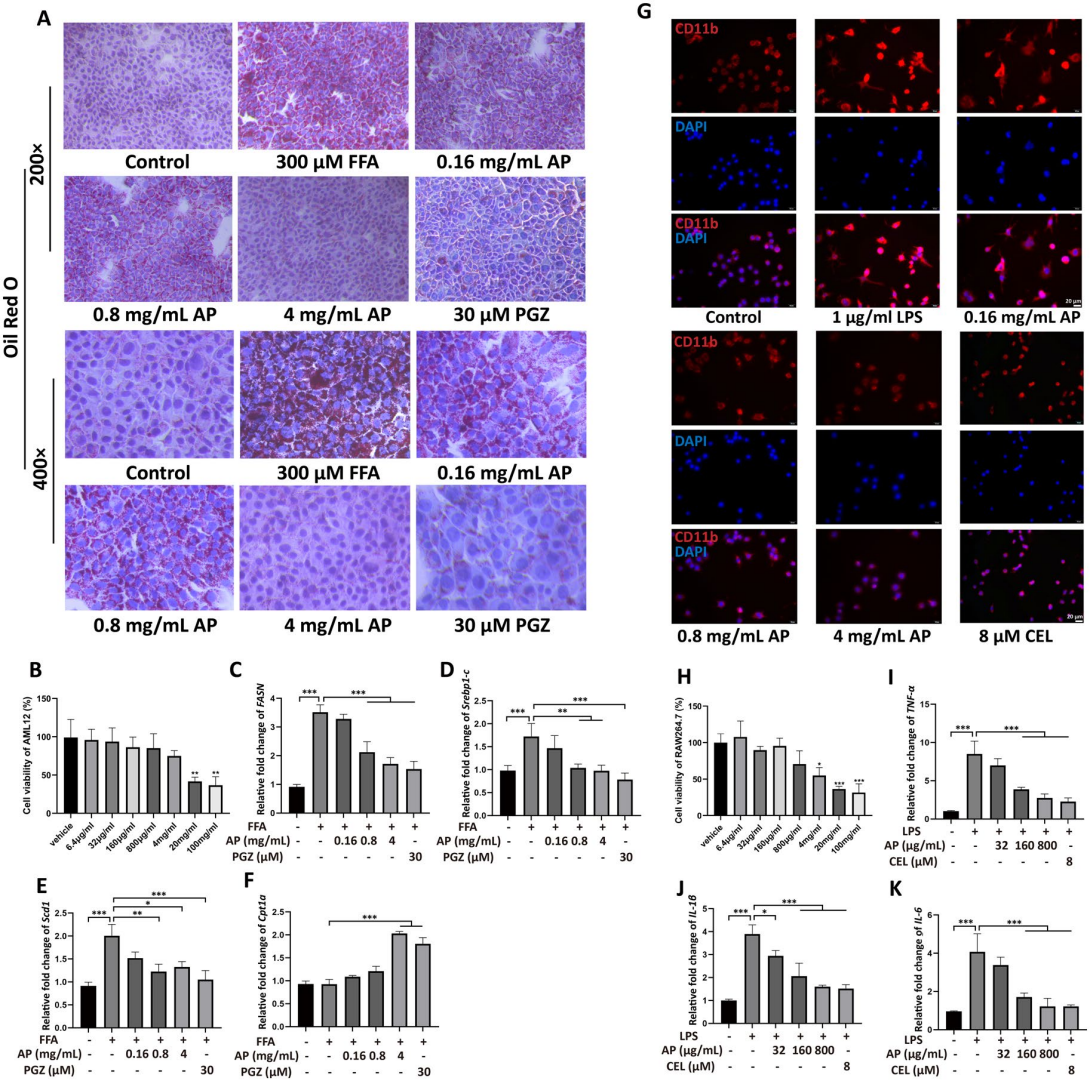
Effects of *A. pilosa* on *In vitro* models of steatosis and inflammation in MASH. Different doses of *A. pilosa* were applied to FFA-induced AML12 cells, with Oil Red O staining used to assess lipid accumulation (magnifications, 200× and 400×) (A). AML12 cell viability was measured (B), along with the mRNA expression levels of lipid metabolism-related genes: FASN (C), SREBP-1c (D), SCD1 (E), and CPT1A (F). Additionally, *A. pilosa* was applied at various doses to LPS-induced RAW264.7 cells, with fluorescence staining to visualize activation markers (CD11b, shown in red, and nuclei stained with DAPI, shown in blue) (G). RAW264.7 cell viability was assessed (H), as well as the mRNA expression levels of inflammation-related genes TNF-α (I), IL-1β (J), and IL-6 (K). Comparisons with the model group are indicated as follows: *p < 0.05, **p < 0.01, ***p < 0.001.

Cell viability analysis using CCK-8 demonstrated that AP at concentrations ≤4 mg/ml exhibited no cytotoxicity in AML12 cells (Figure 3B). A cellular model of hepatic steatosis was established with 300 μM FFA, followed by intervention with AP at 160 μg/ml, 800 μg/ml, or 4 mg/ml. Oil Red O staining revealed that AP at all tested concentrations, as well as PGZ, significantly reduced intracellular lipid droplet accumulation (Figure 3A). RT-PCR analysis indicated that medium and high doses of AP and PGZ significantly downregulated the mRNA expression of SREBP-1c, FASN, and SCD1 (*p* < 0.001, *p* < 0.01, *p* < 0.05), while upregulating CPT1A expression (*p* < 0.001) (Figure 3C–F).

### AP alleviates LPS-induced inflammatory responses in RAW264.7 cells

CCK-8 assay demonstrated that AP at concentrations ≤800 μg/ml did not significantly affect RAW264.7 cell viability (Figure 3H). An *in vitro* model of hepatic inflammatory responses was established using 1 μg/ml LPS., followed by treatment with AP at 32 μg/ml, 160 μg/ml, or 800 μg/ml. Immunofluorescence analysis showed that LPS-stimulated cells exhibited abundant pseudopodia and strong CD11b expression, indicative of macrophage activation. Medium and high doses of AP, as well as CEL, markedly suppressed CD11b expression and inhibited macrophage activation (Figure 3G). RT-PCR results confirmed that AP significantly reduced mRNA expression of TNF-α, IL-1β, and IL-6 compared with the HFHCD group (all *p* < 0.001) (Figure 3I–K).

### Identification of active constituents and putative targets of AP in MASH

**Figure 4.**
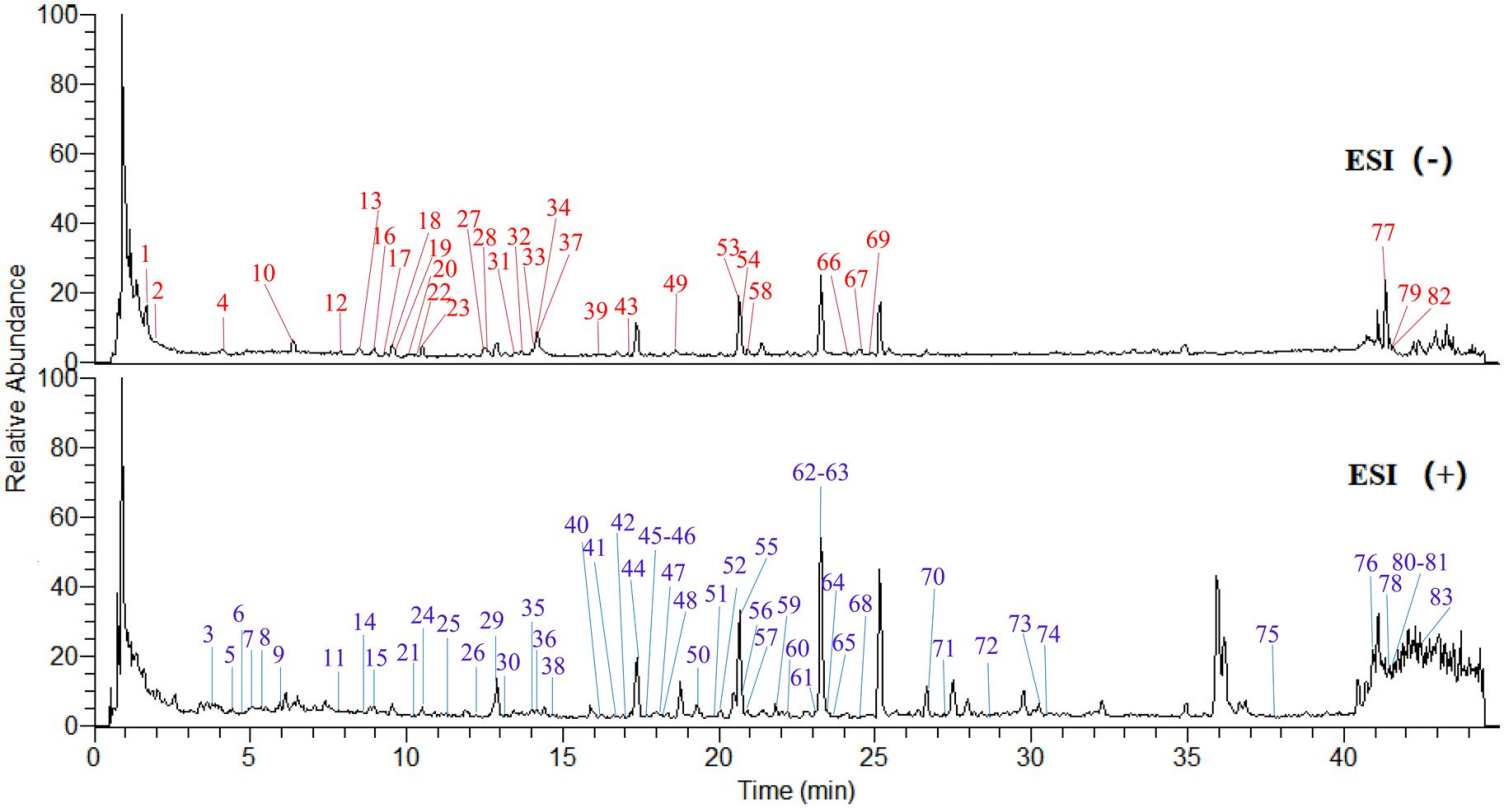
Component identification and fingerprint profiling of *A. pilosa* extract by UHPLC-Q-Exactive Orbitrap HRMS. The upper panel shows results in negative ion mode (ESI^−^), and the lower panel shows results in positive ion mode (ESI^+^).

Using UHPLC-Q-Exactive Orbitrap HRMS, a total of 83 chemical constituents were identified from AP, including 32 compounds in ESI^−^ mode and 51 compounds in ESI^+^ mode, primarily flavonoids, coumarins, and phenylpropanoids (Figure 4; full compound list in Supplementary Table 3).

**Figure 5.**
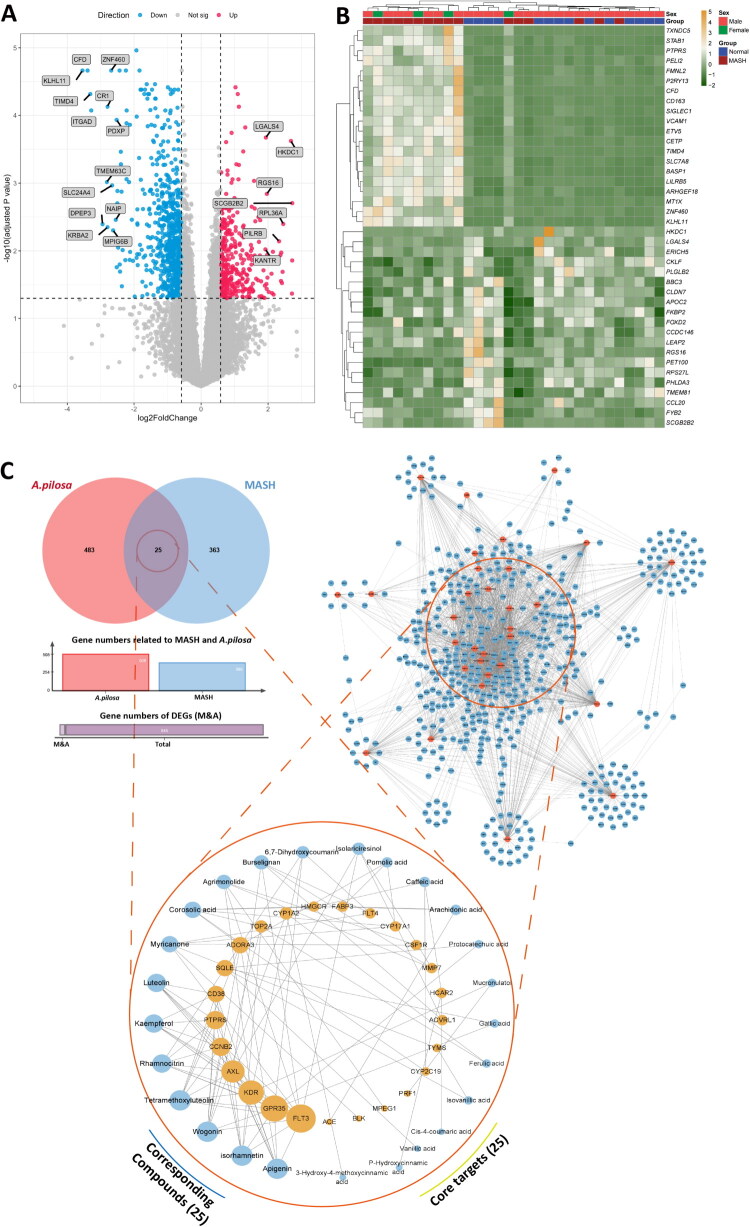
Differentially expressed genes (DEGs) between MASH patients and healthy controls and overlapping genes between DEGs and predicted targets. A volcano plot (A) and heatmap (B) of DEGs illustrate upregulated genes in red and downregulated genes in blue. A network diagram (C) depicts the interaction between key active compounds in *A. pilosa* (green triangles) and core genes relevant to its therapeutic effects in MASH (orange circles).

Transcriptomic dataset GSE126848 from the GEO database, comprising 15 simple fatty liver patients, 16 MASH patients, 12 obese individuals, and 14 healthy controls, was analyzed. Differentially expressed genes (DEGs) between MASH patients and healthy subjects were screened (*p* < 0.01, |log_2_FC| > 1), yielding 388 significant DEGs. Volcano plot highlighted the top 13 downregulated (blue) and top 7 upregulated (red) genes (Figure 5A). Heatmap clustering of the top 20 upregulated genes revealed enrichment in macrophage activation (e.g., STAB1, CD163) and endothelial dysfunction (VCAM1), whereas downregulated genes such as HKDC1 and APOC2 were implicated in lipid accumulation, and LGALS4 in impaired anti-inflammatory responses (Figure 5B).

SwissTargetPrediction was employed to predict 508 putative targets for the identified AP constituents (Figure 5C). Intersection analysis of 508 predicted targets with 388 MASH-associated DEGs identified 25 potential therapeutic targets. The top-ranked targets included FLT3, GPR35, KDR, AXL, CCNB2, PTPRS, CD38, SQLE, ADORA3, and TOP2A (Figure 5C). Network pharmacology analysis revealed that 25 active compounds were closely associated with these 25 targets, as illustrated in the compound–target interaction network (Figure 5C).

### Core targets and active components of AP in MASH treatment

To identify the key therapeutic targets of AP in MASH, this study constructed a protein-protein interaction (PPI) network based on 25 overlapping targets identified from the intersection of predicted compound targets and differentially expressed genes in liver tissues of MASH patients. The network, generated using the STRING database, comprised 23 nodes and 101 edges, indicating a high degree of functional connectivity among these disease-related targets.

**Figure 6.**
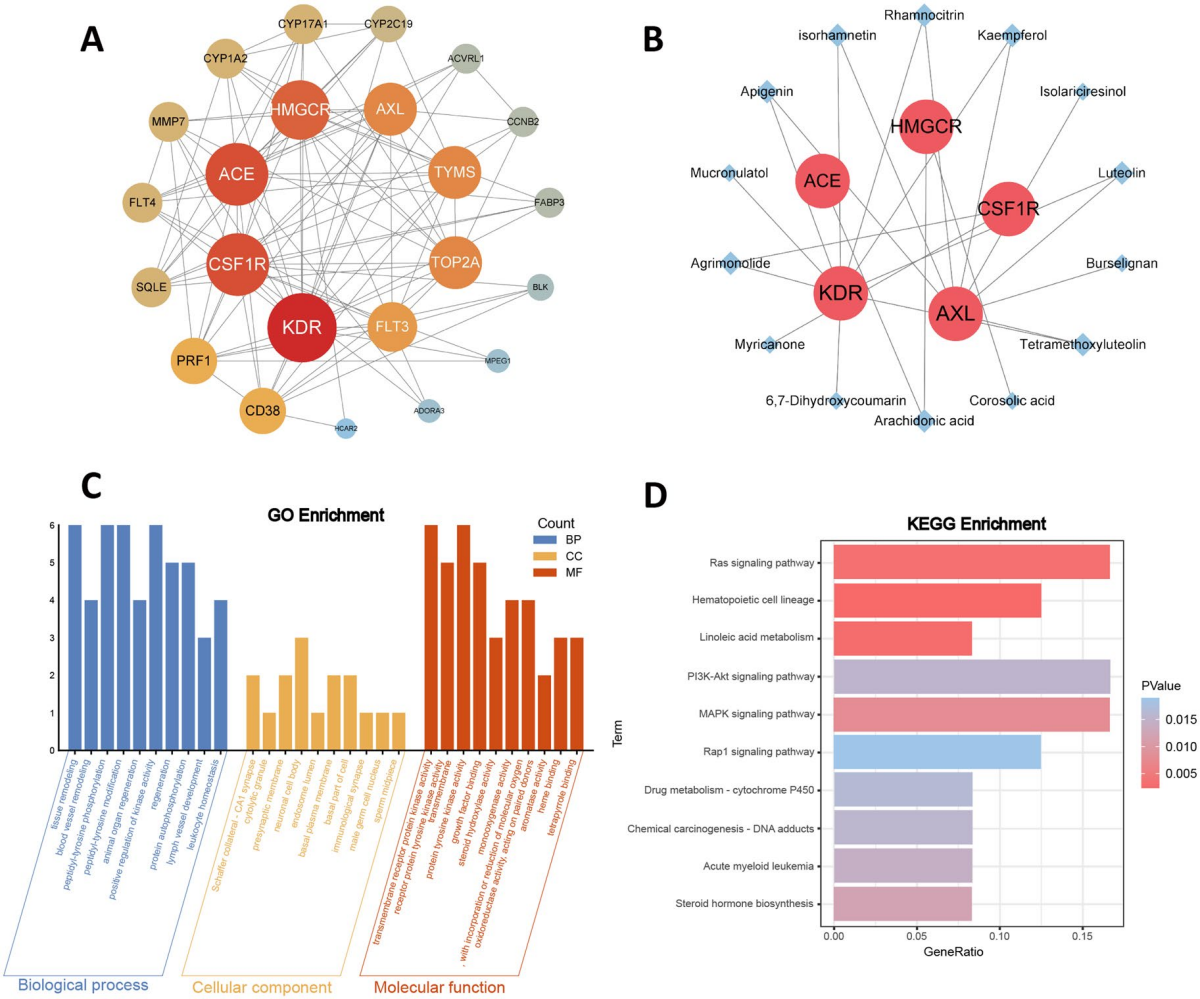
Network pharmacology analysis of *A. pilosa* in the treatment of MASH. (A) Protein-protein interaction (PPI) network of intersecting genes between *A. pilosa* and MASH. (B) Topological network displaying the top 5 core genes (by degree value) and the 15 key active components of *A. pilosa* related to MASH treatment. (C) GO and KEGG enrichment analyses of intersecting genes associated with *A. pilosa* in MASH treatment.

Topological analysis was performed using the CytoNCA plugin, and five hub targets were identified based on degree centrality: KDR, CSF1R, ACE, HMGCR, and AXL (Figure 6A). These proteins exhibited the highest number of direct interactions within the network, suggesting their potential roles as key regulatory nodes connecting multiple signaling pathways. Notably, these hub targets have been previously demonstrated to be closely associated with lipid metabolism, inflammatory signaling, macrophage activation, and vascular dysfunction, which represent critical pathological processes underlying hepatic steatosis and liver inflammation. This association supports their central position in the network and underscores their biological relevance.

Based on compound-target network analysis, eight major active components from AP were further identified: arachidonic acid, kaempferol, luteolin, tetraметhoxyluteolin, rhamnocitrin, agrimonolide, isorhamnetin, and apigenin (Figure 6B). Among these, agrimonolide was identified as the signature component of AP, and its pivotal role is emphasized in subsequent mechanistic analyses.

Gene Ontology (GO) enrichment analysis of the 25 core targets revealed their primary involvement in biological processes including tissue remodeling, leukocyte homeostasis, protein autophosphorylation, kinase activity regulation, and lymphangiogenesis, reflecting coordinated regulation of inflammatory responses and metabolic adaptation. At the molecular function level, enrichment was predominantly observed in protein kinase activity, tyrosine kinase activity, growth factor binding, heme binding, and tetrapyrrole binding. Cellular component analysis indicated primary localization to the basal cell region, presynaptic membrane, endosome lumen, and immunological synapse (Figure 6C; Supplementary Figure 1).

KEGG pathway enrichment analysis further demonstrated that the therapeutic effects of AP against MASH are primarily associated with classical signaling pathways involved in metabolic regulation and inflammation, including the Ras, PI3K-Akt, MAPK, and Rap1 pathways (Figure 6D; Supplementary Figure 1). These signaling cascades do not function as isolated pathways but rather constitute an interconnected mechanistic network that links target modulation to downstream phenotypic outcomes such as amelioration of hepatic steatosis and suppression of liver inflammation, thereby providing a mechanistic framework for subsequent experimental validation.

### Molecular docking and molecular dynamics simulation analysis of representative compounds from AP with core targets

**Figure 7.**
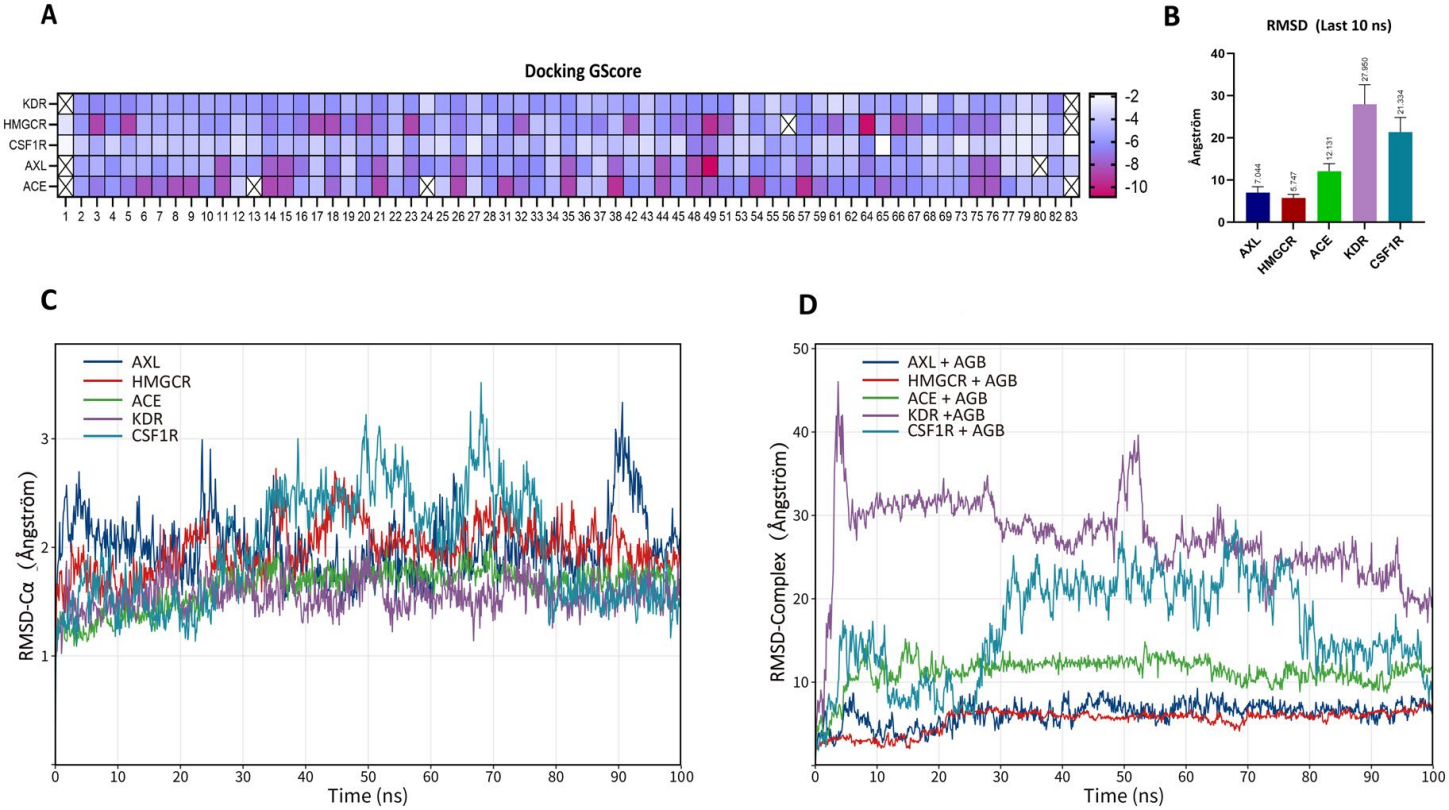
Virtual screening of core components from *A. pilosa* with core targets. (A) Docking interactions of core components from *A. pilosa* with HMGCR, ACE, KDR, AXL, and CSF1R. (B) The average ± standard deviation of RMSD during the last 10 ns of molecular dynamics simulation between AGB and the core targets. (C) The RMSD of the Cα backbone during the molecular dynamics simulation of AGB with the core targets. (D) The RMSD of the ligand-protein complex during the molecular dynamics simulation of AGB with the core targets.

Molecular docking analysis was performed to explore the potential interactions between major constituents of AP and the five core targets identified in the PPI network (Figure 7A). Docking scores were employed as a relative ranking criterion to compare the binding propensity of candidate compounds to the same target, rather than as an absolute measure of binding affinity. Among the tested compounds, agrimol B (AGB) demonstrated consistently superior docking performance across multiple targets, achieving the highest overall docking score at the KDR target. Consequently, AGB was prioritized as the representative bioactive compound for subsequent dynamic simulation analysis.

AGB exhibited favorable docking interactions with HMGCR, ACE, KDR, AXL, and CSF1R, forming reasonable binding conformations within the active sites or regulatory regions of these proteins. To further evaluate the stability and dynamic behavior of the selected protein–AGB complexes, molecular dynamics (MD) simulations were conducted. During the initial MD simulations, root-mean-square deviation (RMSD) values for all protein–AGB complexes remained below 2.5 Å, indicating that the docked systems maintained structural stability throughout the simulation period (Figure 7C,D).

**Figure 8.**
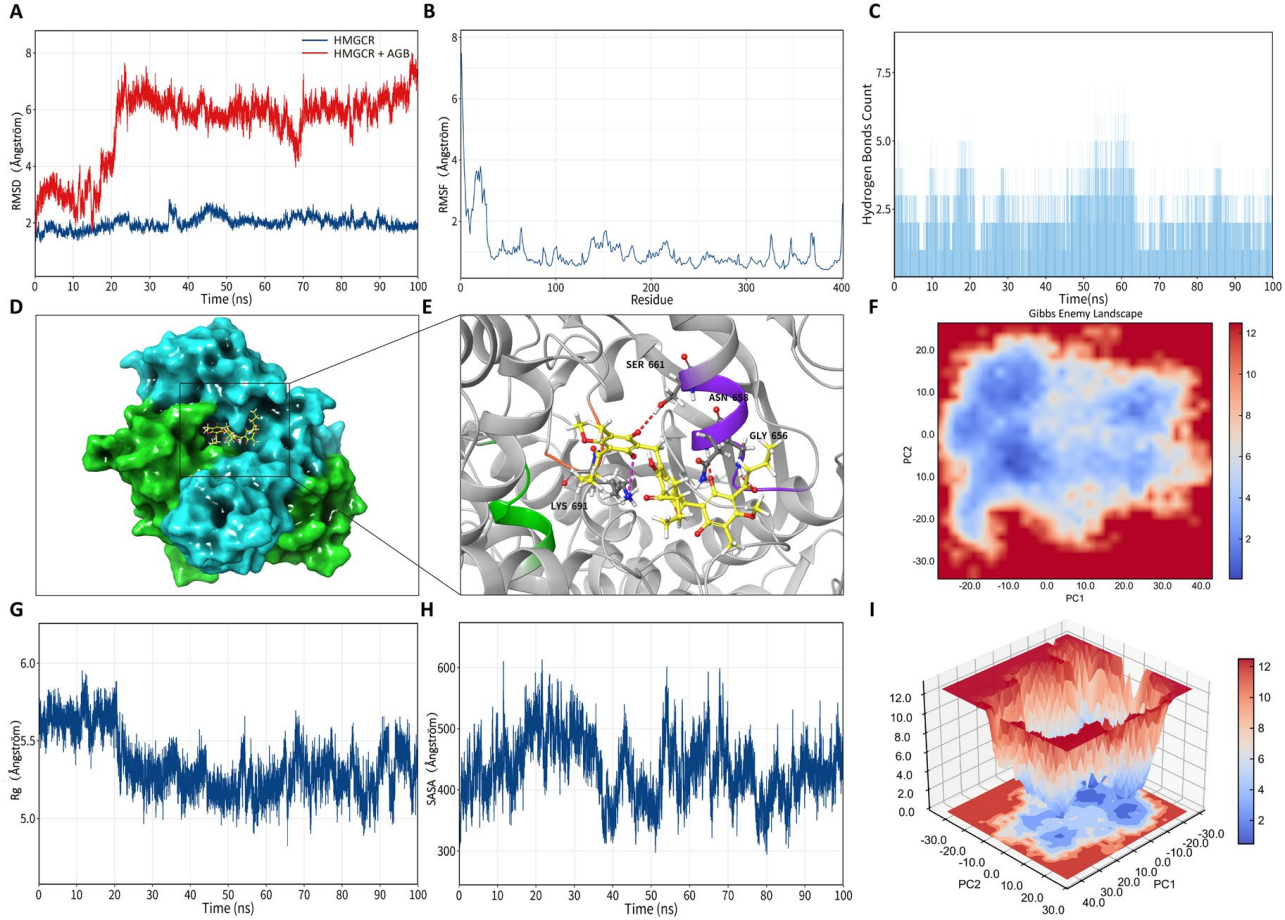
Molecular dynamics simulation of AGB with core targets AXL. (A) RMSD of the AGB–AXL complex. (B) RMSF. (C) Number of hydrogen bonds. (D) Schematic diagram of MM/GBSA-selected conformations. (E) Schematic diagram of ligand–receptor interactions. (F) FEL (free energy landscape) diagram. (G) Radius of gyration (Rg). (H) Solvent accessible surface area (SASA). (I) PCA of the FEL.

**Figure 9.**
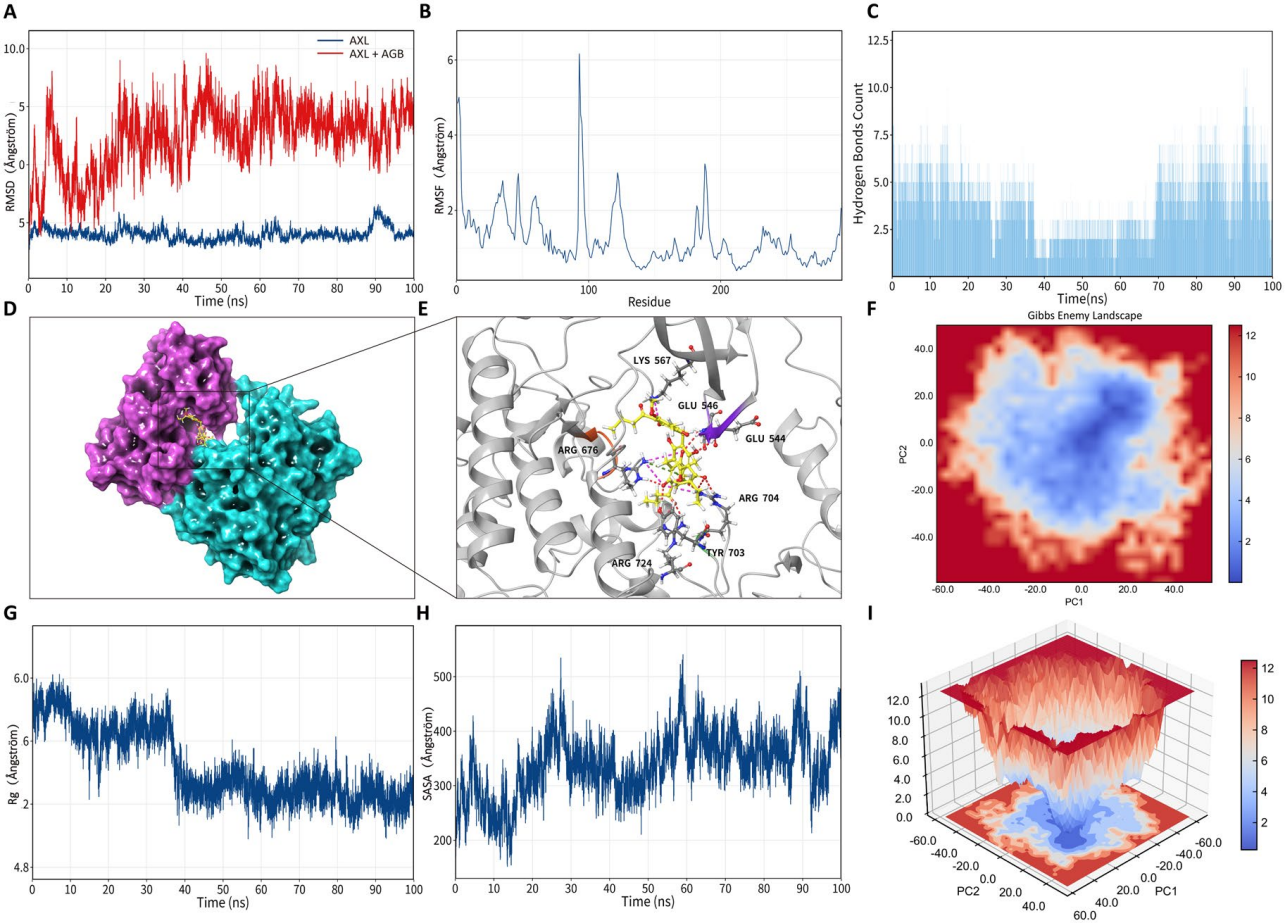
Molecular dynamics simulation of AGB with core targets HMGCR. (A) RMSD of the AGB–HMGCR complex. (B) RMSF. (C) Number of hydrogen bonds. (D) Schematic diagram of MM/GBSA-selected conformations. (E) Schematic diagram of ligand–receptor interactions. (F) FEL (free energy landscape) diagram. (G) Radius of gyration (Rg). (H) Solvent accessible surface area (SASA). (I) PCA of the FEL.

Based on docking performance, biological relevance to lipid metabolism and inflammation, and feasibility of extended simulations, HMGCR and AXL were selected for further 100 ns MD simulations. RMSD trajectories revealed distinct dynamic behaviors between the two complexes: the HMGCR–AGB complex reached a relatively stable conformation after approximately 40 ns, with RMSD values stabilizing around 6–7 Å, whereas the AXL–AGB complex exhibited greater conformational fluctuations, with RMSD values ranging between 6–9 Å (Figures 8A and 9A). These differences suggest target-specific binding dynamics rather than a uniform interaction pattern.

Root-mean-square fluctuation (RMSF) analysis revealed that several residues proximal to the binding region displayed enhanced flexibility, including Lys691 and Ser661 in HMGCR, and Glu544, Arg704, and Tyr703 in AXL (Figures 8B and 9B). Hydrogen bond analysis further confirmed the formation of stable intermolecular interactions throughout the simulations, with the AXL–AGB complex capable of forming up to 12 hydrogen bonds during later stages (Figures 8C–E and 9C–E), supporting sustained ligand–protein interactions despite the presence of conformational flexibility.

Energy landscape analysis demonstrated that the HMGCR–AGB complex possessed a relatively compact and converged free energy distribution, whereas the AXL–AGB complex exhibited a broader and more dispersed energy profile (Figures 8F–I and 9F–I). Consistently, radius of gyration and solvent-accessible surface area (SASA) analyses indicated that the HMGCR–AGB complex adopted a relatively compact conformation over time, while the AXL–AGB complex showed increased solvent exposure, reflecting higher structural flexibility (Figures 8G–H and 9G–H). Collectively, these computational results suggest that AGB may form stable and dynamically reasonable interactions with multiple core targets, particularly HMGCR and AXL.

### Effects of AGB on free fatty acid-induced AML12 *in vitro* model and its regulation of the HMGCR-related signaling pathway

**Figure 10.**
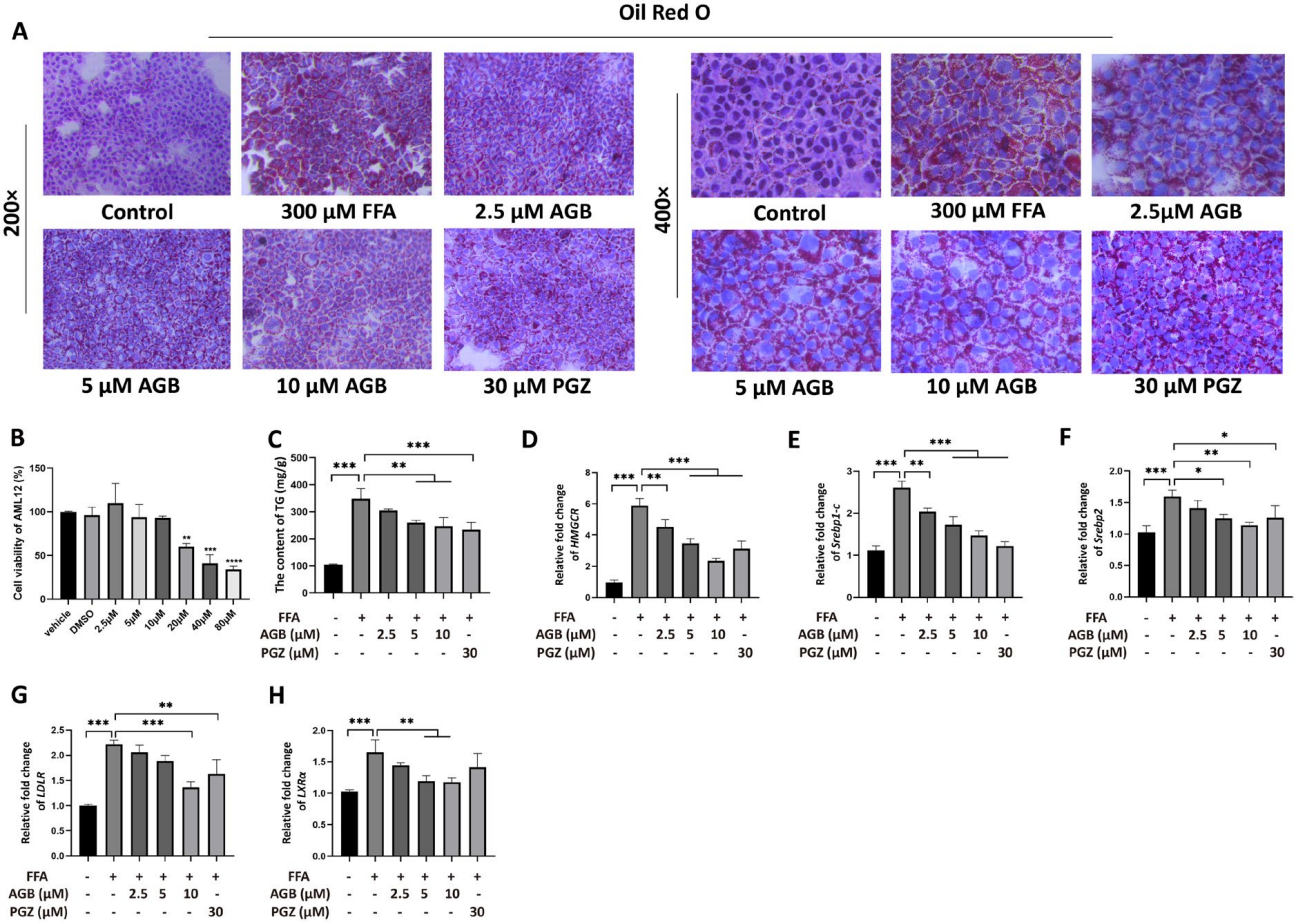
Effect of AGB intervention on FFA-induced AML12 cells and its regulation of HMGCR-related factors. (A) Oil Red O staining, 200× and 400×. (B) CCK-8 assay of AML12 cells. (C) TG content. (D) HMGCR mRNA. (E) Srebp1c mRNA. (F) Srebp2 mRNA. (G) LDLR mRNA. (H) LXRα mRNA.

Cell viability was assessed using the CCK-8 assay to evaluate the cytotoxicity of AGB at different concentrations. The results demonstrated that AGB exhibited no significant cytotoxicity at concentrations of 10 μM or lower (Figure 10B). A cellular model of hepatic steatosis was established using 300 μM FFA, followed by treatment with AGB at concentrations of 2.5 μM, 5 μM, and 10 μM. Oil Red O staining revealed that, compared with the HFHCD group, all doses of AGB as well as the reference drug pioglitazone (PGZ) markedly reduced intracellular lipid droplet accumulation in hepatocytes (Figure 10A). Notably, medium and high doses of AGB significantly decreased intracellular triglyceride (TG) levels (*p* < 0.01).

To further investigate the mechanism, RT-PCR was performed to measure the mRNA expression of genes involved in lipid metabolism. The results showed that, compared with the HFHCD group, medium and high doses of AGB as well as PGZ significantly downregulated the expression of HMGCR, SREBP-1c, SREBP-2, and LXRα (*p* < 0.001; *p* < 0.001; *p* < 0.05; *p* < 0.01, respectively). Moreover, high-dose AGB significantly reduced the mRNA expression of LDLR (*p* < 0.001) (Figure 10C–H).

These findings suggest that AGB may suppress HMGCR expression, thereby reducing cholesterol biosynthetic activity. Concurrently, AGB was observed to interfere with the maturation and nuclear translocation of the transcription factor SREBP-2, consequently attenuating endogenous cholesterol biosynthesis-related signaling pathways and affecting LDLR expression, which may facilitate the clearance of extracellular cholesterol. Additionally, AGB treatment was associated with alterations in the expression of nuclear receptors LXRα and LXRβ, changes that may contribute to enhanced reverse cholesterol transport and bile acid synthesis. Collectively, these regulatory effects are consistent with a mechanism whereby AGB ameliorates intracellular cholesterol accumulation in hepatocytes through modulation of hepatic cholesterol homeostasis.

## Discussion

MASH, the progressive phenotype of MASLD, is characterized by a multifactorial pathogenesis that makes it challenging for single-target drugs to achieve satisfactory clinical efficacy (Geng et al. 2025). In recent years, multi-component and multi-target intervention strategies derived from natural products have attracted increasing attention, owing to their ability to simultaneously modulate metabolic imbalance, inflammatory responses, and fibrogenesis (He et al. 2021; Gouda et al. 2023). The present study demonstrated that *Agrimonia pilosa* Ledeb. (*A. pilosa*, AP) exerts notable protective effects in both *in vivo* MASH animal models and *in vitro* assays. Moreover, through phytochemical profiling, network pharmacology, and molecular simulations, the potential bioactive compounds and their putative targets were identified. These findings not only enrich the pharmacological understanding of traditional herbal medicines but also provide novel insights into the prevention and treatment of MASH.

From a clinical perspective, the development of MASH is closely associated with insulin resistance, dysregulated lipid metabolism, proinflammatory cytokine release, and oxidative stress, which are dynamically intertwined (Noureddin and Sanyal 2018). Many drug candidates previously investigated have targeted a single pathway. For example, the FXR agonist obeticholic acid primarily modulates bile acid metabolism (Durazzo et al. 2025), while the PPAR agonist pioglitazone improves insulin resistance and lipid deposition. However, both agents have shown variable efficacy and safety concerns during long-term clinical use (Devchand et al. 2018). AP is rich in flavonoids (Liu et al. 2014) and phenolic compounds (Kato et al. 2010), which not only directly regulate lipid metabolism-related transcription factors such as SREBPs and LXRs (Li et al. 2023), but also ameliorate the hepatic microenvironment through anti-inflammatory and antioxidant activities (Liao et al. 2024). This enables multi-pathway intervention in disease progression. Of particular interest, our study revealed that AGB, one of the core phenolic compounds, can form stable interactions with key proteins such as HMGCR and AXL, inducing pronounced conformational changes. This provides a structural rationale for its ability to improve hepatic lipid homeostasis and inflammatory responses.

Beyond lipid metabolic dysregulation and inflammation, redox imbalance and mitochondrial dysfunction represent core and interconnected mechanisms driving the progression of hepatic steatosis. The accumulation of excess lipids imposes a substantial metabolic burden on hepatocyte mitochondria, resulting in impaired oxidative phosphorylation, increased electron leakage, and excessive generation of reactive oxygen species (ROS). This redox imbalance promotes lipid peroxidation, mitochondrial DNA damage, and activation of redox-sensitive inflammatory signaling pathways, thereby amplifying hepatocellular injury and inflammatory responses (Valenzuela and Videla 2018; Videla and Valenzuela 2022).

Notably, accumulating evidence suggests that hepatoprotection is not solely attributable to inflammatory suppression, but also involves the coordinated regulation of mitochondrial function, redox balance, and metabolic signaling. Previous studies have demonstrated that interventions capable of maintaining mitochondrial integrity and improving redox balance, such as modulation of antioxidant defense systems and lipid-derived signaling pathways, can effectively prevent the transition from simple steatosis to steatohepatitis-like injury (Valenzuela and Videla 2018). Consistent with this notion, disruption of hepatic redox equilibrium has been recognized as a critical determinant linking metabolic stress, chronic inflammation, and the progression of metabolic liver disease (Videla and Valenzuela 2022).

In this context, AP, enriched in flavonoid and phenolic constituents, may exert hepatoprotective effects through attenuation of oxidative stress and preservation of mitochondrial function, thereby indirectly limiting inflammatory amplification and reinforcing metabolic homeostasis. Such redox-centered mechanisms provide a plausible mechanistic link between our observed improvements in hepatic steatosis and inflammation with AP treatment and its broader modulatory effects on metabolic and immune pathways.

HMGCR, the rate-limiting enzyme in cholesterol biosynthesis, plays a central role in intrahepatic cholesterol accumulation and lipotoxicity when upregulated (Hong et al. 2023). Statins, widely used in clinical practice, inhibit HMGCR to achieve lipid-lowering and cardiovascular benefits. Nevertheless, their application in MASH has been debated due to potential hepatotoxicity and inter-individual variability (Ahsan et al. 2020). As a naturally derived HMGCR regulator, AGB was found not only to reduce HMGCR expression and enzymatic activity but also to suppress the maturation and nuclear translocation of SREBP-2, thereby indirectly modulating LDLR levels. This indicates that AGB acts through multilayered transcriptional regulatory networks rather than simple competitive inhibition, potentially leading to more moderate and sustained metabolic improvements. Such a profile is particularly relevant to MASH patients, who often present with comorbid diabetes, obesity, and cardiovascular disease, where drug tolerability and long-term safety are critical determinants for clinical translation.

Another important observation was the role of AXL within the AGB-target interaction network. AXL, a member of the TAM receptor tyrosine kinase family, has been implicated in hepatic inflammation, fibrosis, and hepatocarcinogenesis (Davra et al. 2016). Hyperactivation of AXL drives macrophage polarization, promotes proinflammatory cytokine secretion, and accelerates fibrogenesis by activating hepatic stellate cells (Grøndal et al. 2024). Our molecular simulations indicated that AGB binding to AXL induces major conformational rearrangements and enhances hydrogen-bonding networks, suggesting its potential as a functional AXL modulator. Given the lack of clinically approved and safe AXL inhibitors (Pyo et al. 2022), AGB represents a promising natural scaffold for future drug development. Validation of AGB’s inhibitory effects on AXL signaling in clinical cohorts may open avenues not only for anti-inflammatory and antifibrotic strategies in MASH but also for preventing progression to hepatocellular carcinoma.

It is noteworthy that the therapeutic activity of AP in alleviating hepatic steatosis and inflammation is not solely attributable to a single compound. Network pharmacology revealed that flavonoids such as kaempferol, luteolin, and isorhamnetin also contribute to modulating lipid synthesis, inflammatory responses, and apoptosis. Kaempferol and luteolin, in particular, have been shown to improve lipid metabolism *via* AMPK signaling (Varshney et al. 2018; Wang et al. 2023) and to alleviate inflammation through NF-κB inhibition (Xia et al. 2014; Bian et al. 2019). This multi-component, multi-target profile underscores the inherent advantage of traditional herbal medicines in treating complex diseases and may explain why crude AP extracts sometimes achieve comparable or superior efficacy relative to isolated compounds in animal models. From a translational perspective, standardized extracts or formulations of AP may be more clinically practical than single-compound drug development.

In addition, systems biology analyses highlighted that AP’s therapeutic effects extend beyond lipid metabolism to immune homeostasis and inflammatory regulation. Differential gene expression analyses revealed that genes associated with macrophage activation, such as STAB1 and CD163, are upregulated in livers from HFD-fed mice exhibiting hepatic steatosis and inflammation, while AP reduced TNF-α, IL-1β, and IL-6 expression in RAW264.7 macrophages. This suggests a role in reshaping the hepatic immune microenvironment to attenuate chronic inflammation, a key driver of progression from simple fatty liver to steatohepatitis-like liver injury and eventually to fibrosis and cirrhosis (Luci et al. 2020). Considering recent insights into the gut–liver axis in steatohepatitis-like liver injury, flavonoid-mediated modulation of gut microbiota composition may also contribute (Sharma et al. 2025), providing new directions for future exploration of AP’s systemic regulatory effects.

In summary, the therapeutic potential of AP and its representative bioactive constituent AGB in hepatic steatosis and liver inflammation can be conceptually summarized as follows: First, modulation of targets associated with cholesterol metabolism, including HMGCR and its related transcriptional regulatory factors, may contribute to improved cholesterol homeostasis and alleviation of lipotoxic stress. Second, regulation of inflammation- and fibrosis-related targets, such as AXL, is associated with attenuation of pro-inflammatory signaling and fibrotic responses. Third, the presence of multiple bioactive constituents suggests a multi-target regulatory paradigm wherein coordinated convergence of metabolic and inflammatory pathways collectively underlies the observed overall protective effects. These multi-target, multi-mechanistic characteristics align with the complex pathophysiological features of hepatic steatosis and liver inflammation, and are consistent with the proposed advantages of botanical medicines in metabolic disease management, whereby comprehensive intervention is achieved through synergistic actions of multiple components.

Future research should focus on the translational potential of AP, including pharmacokinetic profiling to establish exposure–response relationships of its bioactive components, multi-omics approaches to elucidate global regulatory effects on hepatic metabolic networks, and clinical studies to evaluate efficacy and safety in populations with comorbid metabolic syndrome and cardiovascular disease. Given the current lack of approved targeted therapies for MASH worldwide, investigations into AP and its active constituents may provide promising breakthroughs for multi-target interventions.

## Conclusions

This study demonstrates that AP exerts significant therapeutic effects against MASH through multi-target and multi-pathway mechanisms. *In vivo*, AP reduced body weight, liver index, and serum AST, ALT, TG, TC, and LDL-c levels, while alleviating hepatic inflammation, lipid droplet accumulation, and fibrosis. *in vitro*, it suppressed SREBP-1c/FASN/SCD1 expression, promoted CPT1A-mediated fatty acid oxidation, and inhibited macrophage inflammatory responses. Network pharmacology and transcriptomics identified KDR, CSF1R, ACE, HMGCR, and AXL as core targets, while molecular docking and dynamics simulations confirmed the high-affinity binding of AGB to these proteins. Overall, AP, as a natural medicinal resource, holds considerable promise for the prevention and treatment of MASH. Further validation in human-relevant models and clinical settings, along with structural optimization of its active constituents, will be essential to advance its potential toward drug development.

## Supplementary Material

Supplementary Figure 1.tif

Supplementary Materials_revised.docx

## Data Availability

These data are available by sending a request to the corresponding author.
