# Supplementary figures and images for: Multi-target protective effects of Agrimonia pilosa Ledeb. against metabolic dysfunction-associated steatohepatitis in mice
Source: Pharm Biol. 2026 Mar 9;64(1):414–34. doi: 10.1080/13880209.2026.2632359 (PMC12973797; doi:10.1080/13880209.2026.2632359)

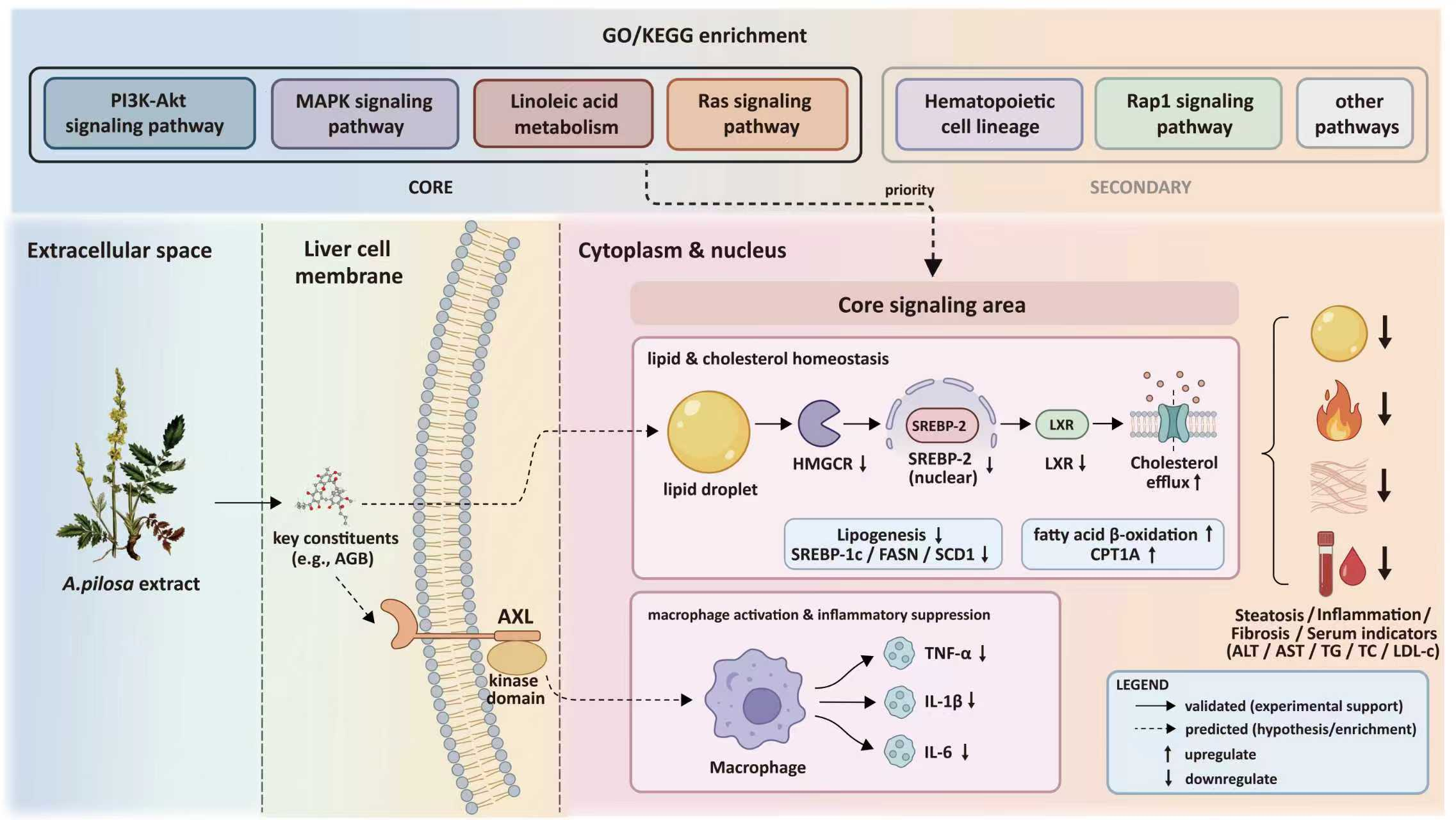

Supplement: Supplementary Figure 1.tif [file IPHB_A_2632359_SM4746.tif]
