# Supplementary material for: Multi-target protective effects of Agrimonia pilosa Ledeb. against metabolic dysfunction-associated steatohepatitis in mice
Source: Pharm Biol. 2026 Mar 9;64(1):414–34. doi: 10.1080/13880209.2026.2632359 (PMC12973797; doi:10.1080/13880209.2026.2632359)
Supplement: Supplementary Materials_revised.docx [file IPHB_A_2632359_SM4745.docx]

Supplementary Materials

**Multi-Target Protective Effects of *Agrimonia pilosa* Ledeb. Against Metabolic Dysfunction-Associated Steatohepatitis in Mice**

**Xinyi Fu^1†^, Jiawen You^1†^, Yunyi Yang^1^, Shenglan Qi^3^, Shiyu Yang^1^, Xiaoxiao Qu^1^, Yanting Shao^1^, Ningwei Wang^1^, Zhiying Wang^1^, Yunhao Li^1^, Min Zheng^1^, Hongjie Yang^1^, Jiajing Zhao^2*^, Xiaoli He^1*^, and Yanming He^1*^**

^1^Department of Endocrinology, Research Laboratory of Pharmacy, Center of Experimental Animals, Clinical Research Institute of Integrative Medicine, Yueyang Hospital of Integrated Traditional Chinese and Western Medicine, Shanghai University of Traditional Chinese Medicine, Shanghai 200437, China;

^2^Shanghai Putuo District Traditional Chinese Medicine Hospital, Shanghai, 200062, China;

^3^Key Laboratory of Liver and Kidney Diseases (Ministry of Education); Institute of Liver Diseases, Shuguang Hospital Affiliated to Shanghai University of Traditional Chinese Medicine, 528 Zhangheng Road, Shanghai, 201203, China;

***Correspondence:**

1. mail: heyanming176@163.com (Yanming He); fishstata@163.com (Xiaoli He); 88460303@qq.com (Jiajing Zhao);

^†^These authors have contributed equally to this work and share first authorship.

**Supplementary Table 1**

|  | Antibodies | Anti-species | Inc. | Dilution | Lot. |
| --- | --- | --- | --- | --- | --- |
| Primary antibodies | CD11b | Mouse | Abcam | 1: 300 | ab8878 |
| Secondary antibodies | Cy3-anti-rabbit | Goat | Abcam | 1: 3000 | ab97035 |

**Table S1.** Information of primary and secondary antibodies.

**Supplementary Table 2**

| Primers | Forward | Reverse |
| --- | --- | --- |
| *CPT1a* | TCCACCGTTTGACTTGTGACCC | CCCTTTATCCATTAGGAGCCGAT |
| *SREBP1c* | AACCTCATCCGCCACCT | GGTAGACAACAGCCGCATC |
| *SCD1* | CAGTTCCTACACGACCACCACTA | GGACGGATGTCTTCTTCCAGAT |
| *FASN* | ACCTCATCACTAGAAGCCACCAG | GTGGTACTTGGCCTTGGGTTTA |
| *TNF-α* | AAAATTCGAGTGACAAGCCTGTAG | CCCTTGAAGAGAACCTGGGAGTAG |
| *IL-1β* | AATCTATACCTGTCCTGTGTAATGAAAGAC | TGGGTATTGCTTGGGATCCA |
| *IL-6* | CCTCTGGTCTCTGGAGTACC | GGAGAGCATTGGAAATTGGGG |
| *HMGCR* | AATAAACCAAACCCCG | GACAGCCAAAAGGAAG |
| *SREBP2* | CAGACCTAGACCTCGC | CTGCTCTTAGCCTCAT |
| *LDLR* | GTCTGCTCCCCAACCT | TCCTCCTCTTTACGCC |
| *LXRα* | ATGTTTCTCCTGATTCTGC | CTCCAACCCTATCCCTAA |
| *GAPDH* | CAACTTTGGCATTGTGGAAGG | ACACATTGGGGGTAGGAACAC |
| *β-ACTIN* | CCCATCTACGAGGGCTAT | TGTCACGCACGATTTCC |

**Table S2.** Real-time PCR primer sequences.

**Supplementary Table 3**

| No. | RT (min) | Identification | Molecular formula | Observed m/z | Adducts |
| --- | --- | --- | --- | --- | --- |
| 1 | 1.68 | Succinic acid | C4H6O4 | 117.0179 | [M-H]^-^ |
| 2 | 2.00 | Gallic acid | C7H6O5 | 169.0132 | [M-H]^-^ |
| 3 | 3.81 | ( 2R,3R) -(+) -Taxifolin | C15H12O7 | 327.0473 | [M+Na]^+^ |
| 4 | 4.11 | Protocatechuic aldehyde | C7H6O3 | 153.0181 | [M-H]^-^ |
| 5 | 4.44 | ( 2S,3S) -(-) -Taxifolin | C15H12O7 | 327.0472 | [M+Na]^+^ |
| 6 | 4.74 | Rhamnocitrin | C16H12O6 | 301.0682 | [M+H]^+^ |
| 7 | 4.99 | Pseudoaspidin | C24H30O8 | 447.1977 | [M+H]^+^ |
| 8 | 5.21 | Rhamnocitrin isomer | C16H12O6 | 301.0890 | [M+H]^+^ |
| 9 | 5.95 | Rhamnocitrin isomer | C16H12O6 | 301.0680 | [M+H]^+^ |
| 10 | 6.39 | Protocatechuic acid | C7H6O4 | 137.0230 | [M-H]^-^ |
| 11 | 7.78 | agrimonolide-6-O-glucopyranoside | C24H28O10 | 499.1549 | [M+Na]^+^ |
| 12 | 7.82 | Guaiacol | C7H8O2 | 123.0438 | [M-H]^-^ |
| 13 | 8.47 | Cis-4-coumaric acid | C9H8O3 | 163.0389 | [M-H]^-^ |
| 14 | 8.66 | Desmethylagrimonolide-6-O-β-D-glucopyranoside | C23H26O10 | 463.1571 | [M+H]^+^ |
| 15 | 8.94 | Agrimonotide-6-O-β-D-glucopyranoside | C24H28O10 | 499.1555 | [M+Na]^+^ |
| 16 | 9.03 | Dihydrocaffeic acid | C9H10O4 | 181.0497 | [M-H]^-^ |
| 17 | 9.34 | 6,7-Dihydroxycoumarin | C9H6O4 | 177.0184 | [M-H]^-^ |
| 18 | 9.55 | Chlorogenic acid | C16H18O9 | 353.0880 | [M-H]^-^ |
| 19 | 9.62 | Caffeic acid | C9H8O4 | 179.0340 | [M-H]^-^ |
| 20 | 10.05 | 4-O-Feruloylquinic acid | C17H20O9 | 367.1037 | [M-H]^-^ |
| 21 | 10.10 | Desmethylagrimonolide-6-O-β-D-glucopyranoside isomer | C23H26O10 | 463.1572 | [M+H]^+^ |
| 22 | 10.19 | Isovanillic acid | C8H8O4 | 167.0339 | [M-H]^-^ |
| 23 | 10.50 | Cryptochlorogenic acid | C16H18O9 | 353.0880 | [M-H]^-^ |
| 24 | 10.51 | 3-Hydroxy-4-methoxycinnamic acid | C10H10O4 | 195.0652 | [M+H]^+^ |
| 25 | 11.36 | Vanillin | C8H8O3 | 153.0546 | [M+H]^+^ |
| 26 | 12.15 | Desmethylagrimonolide-6-O-β-D-glucopyranoside isomer | C23H26O10 | 463.1572 | [M+H]^+^ |
| 27 | 12.52 | P-Hydroxycinnamic acid | C9H8O3 | 163.0390 | [M-H]^-^ |
| 28 | 12.66 | 4-p-Coumaroylquinic acid | C16H18O8 | 337.0932 | [M-H]^-^ |
| 29 | 12.79 | (7S,8S)-3-Methoxy-8,4′-oxyneolignan-3′,4,7,9,9′-pentol | C19H24O7 | 365.1570 | [M+H]^+^ |
| 30 | 13.18 | ( 2R，3R) -( + ) -Taxifolin-3-O-β-D-glucopyranoside | C21H22O12 | 467.1158 | [M+H]^+^ |
| 31 | 13.62 | Kaempferol-3-O-α-L-rhamnopyranoside | C21H20O10 | 431.0938 | [M-H]^-^ |
| 32 | 13.69 | 3-O-Feruloylquinic acid | C17H20O9 | 367.1036 | [M-H]^-^ |
| 33 | 13.91 | 5-p-Coumaroylquinic acid | C16H18O8 | 337.0910 | [M-H]^-^ |
| 34 | 14.05 | Vanillic acid | C8H8O4 | 167.0340 | [M-H]^-^ |
| 35 | 14.14 | Quercitrin | C21H20O11 | 449.1063 | [M+H]^+^ |
| 36 | 14.18 | Ferulic acid | C10H10O4 | 195.0652 | [M+H]^+^ |
| 37 | 14.20 | Caffeic acid isomer | C9H8O4 | 179.0340 | [M-H]^-^ |
| 38 | 14.61 | Kaempferol-3-O-β-D-glucopyranoside | C21H20O11 | 449.1051 | [M+H]^+^ |
| 39 | 16.01 | (7R,8S)-4,7,9,9'-Tetrahydroxy-3,3'-dimethoxy-8-O-4'-neolignan-7-O-β-D-glucoside | C26H36O12 | 539.2138 | [M-H]^-^ |
| 40 | 16.09 | 3-Methoxy-8,4'-oxyneolignan-3',4,7,9,9'-pentol | C19H24O7 | 365.1577 | [M+H]^+^ |
| 41 | 16.77 | (7S,8R)-1-[4-O-(β-D-Glucopyranosyl)-3-methoxyphenyl]-2-[4-(3-hydroxypropyl)-2,6-dimethoxyphenoxy]-1,3-propanediol | C27H38O13 | 593.2202 | [M+Na]^+^ |
| 42 | 17.00 | Isopilosanol A or Pilosanol A | C29H32O10 | 563.1895 | [M+Na]^+^ |
| 43 | 17.13 | Tetramethoxyluteolin | C19H18O6 | 341.1007 | [M-H]^-^ |
| 44 | 17.57 | Agrimonolide-6-O-glucopyranoside | C24H28O10 | 499.1570 | [M+Na]^+^ |
| 45 | 17.65 | Burselignan | C20H24O6 | 383.1461 | [M+Na]^+^ |
| 46 | 17.68 | 4,7,9,9'-Tetrahydroxy-3,3'-dimethoxy-8-O-4'-neolignan | C20H26O7 | 401.1568 | [M+Na]^+^ |
| 47 | 17.90 | (+)-4,9,9'-Trihydroxy-3-methoxy-3',7-epoxy-8,4'-oxyneolignan | C19H22O6 | 347.1462 | [M+H]^+^ |
| 48 | 18.07 | Densispicoside | C27H38O13 | 593.2195 | [M+Na]^+^ |
| 49 | 18.75 | Isochlorogenic Acid A | C25H24O12 | 515.1198 | [M-H]^-^ |
| 50 | 19.24 | Icariol A2 | C22H28O9 | 459.1623 | [M+Na]^+^ |
| 51 | 19.75 | Luteolin | C15H10O6 | 287.0525 | [M+H]^+^ |
| 52 | 20.06 | (7S,8S)-Threo-4,7,9,9'-tetrahydroxy-3,3',5'-trimethoxy-8-O-4'-neolignan | C21H28O8 | 431.1673 | [M+Na]^+^ |
| 53 | 20.68 | Ziyu-glucoside II | C35H56O8 | 603.3898 | [M-H]^-^ |
| 54 | 20.71 | Apigenin-7-O-β-D-butylglucuronate | C25H26O11 | 501.1379 | [M-H]^-^ |
| 55 | 20.73 | Ciwujiatone | C22H26O9 | 457.1467 | [M+Na]^+^ |
| 56 | 20.87 | Agrimol E | C33H38O12 | 627.2410 | [M+H]^+^ |
| 57 | 20.91 | Isoquercitrin | C21H20O12 | 465.1066 | [M+H]^+^ |
| 58 | 20.96 | (7R,8R,7′E)-4,7,9,3′,9′-pentahydroxy3-methoxy-8,4′-oxyneolign-7′-ene-3′-O-β-D-glucopyranoside | C25H32O13 | 539.1727 | [M-H]^-^ |
| 59 | 21.73 | Isolariciresinol | C20H24O6 | 383.1463 | [M+Na]^+^ |
| 60 | 22.37 | (+)-4',7-Epoxy-4,9,9'-trihydroxy-3,3',5'-trimethoxy-8,5'-neolignan | C21H26O7 | 391.1725 | [M+H]^+^ |
| 61 | 23.22 | Wogonin | C15H12O5 | 273.0731 | [M+H]^+^ |
| 62 | 23.39 | Kaempferol | C15H10O6 | 287.1251 | [M+H]^+^ |
| 63 | 23.39 | Dihydroxydehydrodiconiferyl alcohol | C20H24O6 | 383.1464 | [M+Na]^+^ |
| 64 | 23.47 | Hyperin | C21H20O12 | 465.1066 | [M+H]^+^ |
| 65 | 23.58 | Agrimol F | C34H40O12 | 641.2580 | [M+H]^+^ |
| 66 | 24.06 | (-)-Evofolin B | C18H20O6 | 331.1167 | [M-H]^-^ |
| 67 | 24.52 | isorhamnetin | C16H12O7 | 315.0489 | [M-H]^-^ |
| 68 | 24.53 | Apigenin | C15H10O5 | 293.0393 | [M+Na]^+^ |
| 69 | 24.95 | Mucronulatol | C17H18O5 | 301.1057 | [M-H]^-^ |
| 70 | 26.50 | 4,7,9,9'-Tetrahydroxy-3,3',5'-trimethoxy-8-O-4'-neolignan | C21H28O8 | 431.1673 | [M+Na]^+^ |
| 71 | 27.18 | ( +)-4″,4‴-dihydroxy-3,3′,3″,3‴,5,5′-hexamethoxy-7,9′;7′,9-diepoxy-4,8″;4′,8‴-bisoxy-8,8′-dineolignan-7″,7‴,9″,9‴-tetraol | C42H50O16 | 833.2972 | [M+Na]^+^ |
| 72 | 28.73 | pilosaneolignan ester | C18H18O5 | 315.1200 | [M+H]^+^ |
| 73 | 30.37 | Agrimonolide | C18H18O5 | 315.1201 | [M+H]^+^ |
| 74 | 30.48 | Kaempferide-3-O-α-L-rhamnopyranoside | C22H22O10 | 469.1102 | [M+Na]^+^ |
| 75 | 37.87 | Dehydrodicatechin A | C30H24O12 | 577.1340 | [M+H]^+^ |
| 76 | 40.97 | Dehydrodicatechin A isomer | C30H24O12 | 577.1335 | [M+H]^+^ |
| 77 | 41.19 | Myricanone | C21H24O5 | 355.1553 | [M-H]^-^ |
| 78 | 41.42 | 1β，2α，3β，19α-terahydroxyurs-12-en-28-oic acid | C30H48O6 | 505.3510 | [M+H]^+^ |
| 79 | 41.65 | Arachidonic acid | C20H32O2 | 303.2330 | [M-H]^-^ |
| 80 | 41.67 | Corosolic acid | C30H48O4 | 473.3618 | [M+H]^+^ |
| 81 | 41.67 | 1β，2β，3β，19α-terahydroxyurs-12-en-28-oic acid | C30H48O6 | 505.3496 | [M+H]^+^ |
| 82 | 41.69 | Agrimol B | C37H46O12 | 681.2962 | [M-H]^-^ |
| 83 | 42.48 | Pomolic acid | C30H48O4 | 473.3618 | [M+H]^+^ |

**Table S3.** Compounds identified in *Agrimonia pilosa* Ledeb.

**Supplementary Table 4**

| **Component** | **Value** |
| --- | --- |
| Moisture (%) | ≤ 10.0 |
| Crude protein (%) | ≥ 20.5 |
| Crude fat (%) | ≥ 4.0 |
| Crude fiber (%) | ≤ 5.0 |
| Crude ash (%) | ≤ 8.0 |
| Calcium (%) | 1.0–1.8 |
| Total phosphorus (%) | 0.6–1.2 |
| Lysine (%) | ≥ 1.32 |
| Methionine + cystine (%) | ≥ 0.78 |
| Sodium chloride (%) | 0.4 |
| Carbohydrate (NFE, %) | 52.5 |
| Energy density (kcal/g) | 3.28 |

**Table S4.** Diet composition of the normal diet. The normal diet (Cat. No. P1101F-25) was purchased from Shanghai Puluteng Biotechnology Co., Ltd. The macronutrient energy distribution was as follows: protein, 25.0%; fat, 11.0%; and carbohydrate, 64.0% of total energy.

**Supplementary Table 5**

| **Ingredient** | **Amount (g)** | **Energy (kcal)** |
| --- | --- | --- |
| Casein | 200 | 800 |
| L-Cystine | 3 | 12 |
| Fructose | 200 | 800 |
| Maltodextrin | 100 | 400 |
| Sucrose | 100 | 400 |
| Cellulose | 50 | 0 |
| Soybean oil | 25 | 225 |
| Lard | 20 | 180 |
| Primex shortening | 135 | 1215 |
| Mineral mix (S10026B) | 50 | 0 |
| Vitamin mix (V10001C) | 1 | 4 |
| Choline bitartrate | 2 | 0 |
| Cholesterol | 18 | 0 |
| FD&C Yellow Dye #5 | 0.05 | 0 |
| Total | 904.05 | 4036 |

**Table S5.** Macronutrient composition and energy distribution of the high-fat–high-fructose– high-cholesterol diet (HFHCD). The HFHCD (Cat. No. XT301) was purchased from Jiangsu Xietong Biotechnology Co., Ltd. Detailed ingredient formulation and energy information, including macronutrient composition and energy density, are provided in this table.

**Supplementary Table 6**

| **Item** | **Amount (g/100 g diet)** | **Energy distribution (%kcal)** |
| --- | --- | --- |
| Protein | 22.50 | 20.0 |
| Fat | 19.90 | 40.0 |
| Carbohydrate | 44.90 | 40.0 |
| Energy density (kcal/g) | 4.46 | Provided by manufacturer |

**Table S6.** Macronutrient composition and energy distribution of the HFHCD. The table summarizes the amounts of protein, fat, and carbohydrate (g per 100 g of diet) and their respective contributions to total energy intake (% kcal).

**Supplementary Table 7**

| **Aspect** | **Normal diet (P1101F-25)** | **HFHCD diet (XT301)** |
| --- | --- | --- |
| Energy density (kcal/g) | 3.28 | 4.46 |
| Protein (%kcal) | 25.0 | 20.0 |
| Fat (%kcal) | 11.0 | 40.0 |
| Carbohydrate (%kcal) | 64.0 | 40.0 |
| Fat source | Low-fat, undefined mixed fats | Soybean oil, lard, shortening |
| Sugar source | Complex carbohydrates (NFE) | Fructose, sucrose, maltodextrin |
| Cholesterol | Not added | 18 g added |
| Fiber source | Natural grain fiber | Purified cellulose |
| Micronutrients | Naturally present in chow | Defined vitamin/mineral mixes |

**Table S7.** Structural differences between the two diets.
